# Integration of relational and hierarchical network information for protein function prediction

**DOI:** 10.1186/1471-2105-9-350

**Published:** 2008-08-22

**Authors:** Xiaoyu Jiang, Naoki Nariai, Martin Steffen, Simon Kasif, Eric D Kolaczyk

**Affiliations:** 1 Department of Mathematics and Statistics, Boston University, Boston, MA 02215, USA; 2 Bioinformatics Program, Boston University, Boston MA, 02215, USA; 3 Department of Genetics and Genomics, Boston University, Boston MA, 02118, USA; 4 Department of Biomedical Engineering, Boston University, Boston MA, 02215, USA

## Abstract

**Background:**

In the current climate of high-throughput computational biology, the inference of a protein's function from related measurements, such as protein-protein interaction relations, has become a canonical task. Most existing technologies pursue this task as a classification problem, on a term-by-term basis, for each term in a database, such as the Gene Ontology (GO) database, a popular rigorous vocabulary for biological functions. However, ontology structures are essentially hierarchies, with certain top to bottom annotation rules which protein function predictions should in principle follow. Currently, the most common approach to imposing these hierarchical constraints on network-based classifiers is through the use of transitive closure to predictions.

**Results:**

We propose a probabilistic framework to integrate information in relational data, in the form of a protein-protein interaction network, and a hierarchically structured database of terms, in the form of the GO database, for the purpose of protein function prediction. At the heart of our framework is a factorization of local neighborhood information in the protein-protein interaction network across successive ancestral terms in the GO hierarchy. We introduce a classifier within this framework, with computationally efficient implementation, that produces GO-term predictions that naturally obey a hierarchical 'true-path' consistency from root to leaves, without the need for further post-processing.

**Conclusion:**

A cross-validation study, using data from the yeast *Saccharomyces cerevisiae*, shows our method offers substantial improvements over both standard 'guilt-by-association' (i.e., Nearest-Neighbor) and more refined Markov random field methods, whether in their original form or when post-processed to artificially impose 'true-path' consistency. Further analysis of the results indicates that these improvements are associated with increased predictive capabilities (i.e., increased positive predictive value), and that this increase is consistent uniformly with GO-term depth. Additional *in silico *validation on a collection of new annotations recently added to GO confirms the advantages suggested by the cross-validation study. Taken as a whole, our results show that a hierarchical approach to network-based protein function prediction, that exploits the ontological structure of protein annotation databases in a principled manner, can offer substantial advantages over the successive application of 'flat' network-based methods.

## Background

Proteins are fundamental to the complex molecular and biochemical processes taking place within organisms. An understanding of their role is therefore critical in biology and bio-related areas, for purposes ranging from general knowledge to the development of targeted medicine and diagnostics. High-throughput sequencing technology has identified a tremendous number of genes with no known functional annotation. On average, as many as 70% of the genes in a genome have poorly known or unknown functions [1]. Not surprisingly, therefore, the prediction of protein function has become an important and urgent problem in functional genomics.

Protein function prediction can take many forms. The traditional and most popular methodologies use homology modeling and sequence similarity to infer biochemical function [2,3]. In simple cases, such as certain families of ribosomal proteins, globins, kinases or caspases, these procedures work reasonably well. Sequence similarity has been used with great success for inference of molecular function. For biological process and pathway annotation, guilt by association using functional linkage methods has been a popular choice in recent years.

For example, microarrays are often used to cluster proteins into groups of genes that respond concordantly to a given environmental stimuli. When these groups are strongly enriched in proteins in a given biological process such as insulin signaling and also contain proteins without annotation we often take the leap of faith and predict the unknown proteins to be associated with this process as well. Similarly, when two proteins are found to interact in a high throughput assay we also tend to use this as evidence of functional linkage.

However, enrichment and guilt by association are often highly misleading and can lead to a very high false positive rate if not used with caution. The work in [4] and several other papers, e.g., [5-7], attempted to frame these inference problems in a precise network-based probabilistic framework. Here we attempt to make a fundamental advance in this area, by augmenting the network-based perspective to additionally make explicit use of the structure of the GO hierarchy to compute more precise probabilities, thereby improving on the quality of predictions made by the inference algorithms.

More broadly, the work in this paper is important in demonstrating that an important role can be played in this context by the knowledge captured in biological ontologies, when properly harnessed. That this should be the case is not obvious *a priori*. For example, while many scientists use GO in their daily research, it can be (and has been) claimed that overlap among categories, as well as the inherent ambiguity and semantic complexity of naming biological functions and processes, can frequently lead to misleading interpretations and wild goose chases. Classic statistical approaches are based on flat disjoint categories, and quantitative measures of annotation similarity such as through semantic similarity remain somewhat *ad hoc*.

Nevertheless, despite such concerns, our work here shows that in the present context of automated protein function prediction, the leverage of hierarchies grounded in biological ontologies can yield real, quantifiable advantages over 'flat' network-based approaches.

### Objective

Computational protein function prediction is typically treated as a classification problem. From this perspective, given a protein *i *and the label *G *of a potential function for that protein, the goal is to predict whether or not *i *has label *G*, using a classifier built from a set of training cases and additional related data. Such related data can be of many types (e.g., protein interaction data, gene expression data, protein localization data) but often can be summarized in the form of a functional linkage graph (e.g., protein-protein interaction network, gene association network). The labels *G *typically derive from a database of terms.

**Figure 1:**
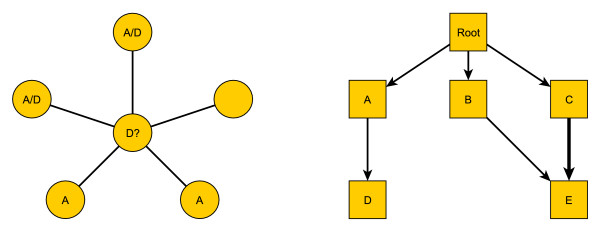
**Visualization small PPI network and GO DAG**. This plot contains two toy examples of Protein-Protein Interaction network and the Gene Ontology structure. (a) Schematic network of local protein interactions; (b) schematic GO hierarchy, where the thicker link indicates larger weight. Among the neighbors of the central protein in (a), 4 out of 5 are labeled with term *A*; 2 out of 5 are labeled with term *D*. One neighbor is not labeled with any term. We want to predict whether or not the central protein has term *D*.

Protein-protein interaction (PPI) data are common, and have been used widely in the protein function prediction problem. A functional linkage graph is used to represent the information in the PPI, where nodes represent proteins and edges indicate pairwise interactions, as in Fig. 1(a). Numerous studies have demonstrated that proteins sharing similar functional annotations tend to interact more frequently than proteins which do not share them, both as members in relatively fixed complexes, e.g. the ribosome, or as transient interactors, such as kinases and substrates in signal transduction networks. Hence, it is natural to want to take advantage of the neighborhood information to predict protein functions. For example, to predict for the protein with a question mark in the center in Fig. 1(a), we can try to utilize any known functional annotations of its neighbors, i.e., the proteins directly interacting with it.

Databases of labels *G *are commonly structured in a hierarchical form (more formally, as a directed acyclic graph (DAG)). The Gene Ontology (GO) database is one such example . Viewed as a DAG, nodes represent labels and a link represents the *is_a *and *part_of *relations between labels. Function assignments to proteins must obey the *true-path rule*: if a child term (i.e., more specific term) describes the gene product, then all its parent terms (i.e., less specific terms) must also apply to that gene product. Fig. 1(b) shows a small schematic illustration. For any protein labeled with term *A*, it may or may not have term *D*, the child of *A*; on the other hand, if it does not have term *A*, it surely does not have *D*.

This annotation rule suggests that when predicting the label of a term in the hierarchy, it is helpful to first consider whether the protein has the parent term or not. Thus, informative are not only the neighbors labeled with the term of interest but also those labeled with the parent. For instance, to predict the label of term *D *for the central protein in Fig. 1(a), we want to use both the neighbors labeled with *A *and the neighbors labeled with *D*. The exploitation of GO hierarchy is not novel, and indeed is natural. It has been used in functional annotation of genes, as mentioned in the Related Work section, as well as for other purposes, such as identifying over- and under-representation of GO terms in a gene dataset, and clustering functionally related genes [8-10].

As currently practiced in most instances, prediction of protein function is done with classifiers trained separately for each possible label *G*, as in [4,7,11,12]. (Please also see the section of Related Work.) But, as just discussed, the overall collection of labels to be assigned generally has a *hierarchical *structure to it i.e., the labels are related to each other in a specific manner. This structure typically is enforced only after producing an initial set of predictions, as post-processing steps, either using transitive closure, [4], or using more sophisticated methods, [13,14].

**Figure 2:**
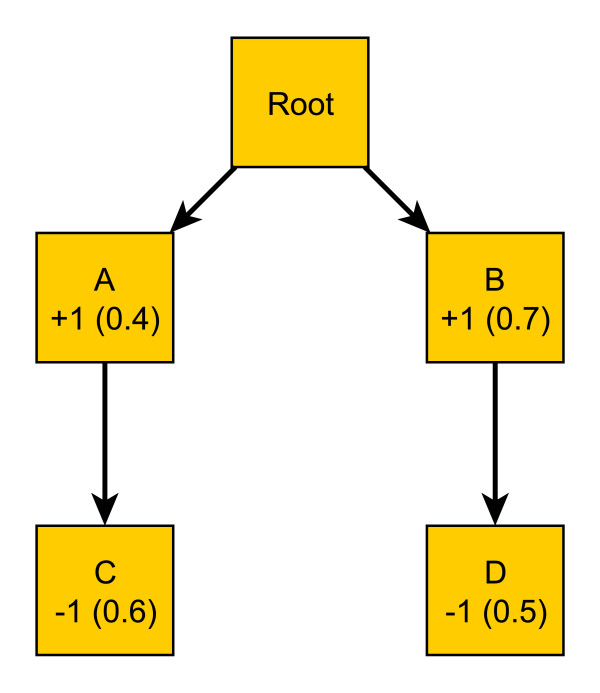
**Illustration of obedience and disobedience to the true-path rule**. The plot demonstrates a small example of GO hierarchy with four terms A, B, C and D. The true annotations and the predicted probabilities of the terms for some protein are also given, in a format of "true annotation (probability)". We use this to illustrate predictions that are consistent and are not consistent with the *the true-path rule*.

To further illustrate this, we show a toy GO hierarchy in Fig. 2, which contains a root and four descendant terms A, B, C and D, where term A and B are the parents for C and D, respectively. For a given protein, the label format for the terms is "true label (predicted probability)". For example, the protein is annotated with term A but not with term D. The probabilities of having term A and D are 0.4 and 0.5, respectively.

Most existing methods, as discussed earlier, predict protein function in a term-by-term fashion, without considering the relationship among terms. Suppose the probabilities in the plot are obtained from one of such methods. If we apply a cut-off of 0.5, which is a commonly used threshold in this field, we will predict that the protein is NOT annotated with term A, since the probability of having A is 0.4, less than 0.5; and is annotated with A's child C. This violates *the true-path rule*, since if the protein is predicted not having term A, then it is not having any of A's descendent terms. On the other hand, the protein is predicted to be labeled with both terms B and D, with probabilities of 0.7 and 0.5, respectively, which obeys *the true-path rule*, with the prediction on D as a false positive. Such a violation to *the true-path rule *is not uncommon.

The basic premise of this paper is that reference to this hierarchical relationship among labels is best incorporated in the initial stage of constructing a classifier, as a valuable source of information in and of itself. Our objective here is to demonstrate the power of this premise and to show that it may be tapped in the form of a single, coherent probabilistic classifier. In particular, we develop a probability model that integrates relational data and a hierarchy of labels, and illustrate its advantages in predicting protein function using a PPI network and the Gene Ontology (GO) hierarchy.

### Related Work

Many methodologies have been proposed to predict protein functions. Most of the earlier methods tend to use a single source of protein information, such as PPI. Typical examples include the "Nearest-Neighbor" algorithm, also known as "guilt-by-association" principle, and the Binomial-Neighborhood (BN) method [4].

These earlier methods were followed later by a surge of interest in combining heterogeneous sources of protein information. For example, a machine learning approach integrating datasets of PPI, gene expression, hydropathy profile and amino acid sequences, in the form of different kernels, has been introduced [11]. Various genome-wide data can also be employed in a Bayesian framework to produce posterior probability for function assignments [5,6]. And a Markov Random Field model combining PPI network and protein domain information was introduced in [12]. A common characteristic of these methods is detecting protein functions individually, without considering the relationship among them. As remarked, a pitfall of this is that the predictions may conflict with the *true-path rule *of ontologies.

Motivated in part by seminal work of [15], combining protein data and ontology structure has recently become a focus. One approach is using a Bayesian network structure to correct inconsistent function predictions, by calculating the largest posterior probability of the *true-path *consistent labels, given the predictions from independent classifiers for each of the proteins [13]. Similar work has been done in [14], where multiple classifiers are built and training data are modified according to the GO hierarchy. A Bayesian model consisting of a set of nested multinomial logit models, where a prior describing correlations of parameters for nearby GO terms is trained by the hierarchy, has been proposed in [16]. Observing the fact that a protein is actually associated with multiple GO terms, this problem can also be treated as a hierarchical multi-label classification task [13,17]. Yielding various degrees of improvement in prediction accuracy, these methods all seek to take advantage of the hierarchical label structure. However, importantly, we note that all of those that predict at multiple depths in the GO hierarchy take a separate step to correct *true-path *inconsistent predictions, rather than producing them directly in a probabilistically coherent fashion.

In summary, combining relational protein data, such as PPI, and hierarchical structures, as in GO, in one probabilistic model to predict *true-path *consistent function labels, has to the best of our knowledge not been done to date. This task is the focus of our work.

## Methods

Ontologies like GO are structured as directed acyclic graphs (DAG's), where a child term may have multiple parent terms. The DAG structure, with alternative paths from the root to internal and leaf terms, is one of the reasons that formal approaches to annotation predictions have been difficult. It is well known that computing the most likely assignment of values to variables in a DAG of size *N *given their conditional probabilities on the arcs is a classical NP-hard problem in graphical models. In fact, variants of this problem are actually formally harder by some theoretical considerations. Therefore, people routinely use tree approximations of probability distributions, which goes back to the work in [18]. In our work, clearly, a tree-based approach is the first step to something concrete, rather than *ad hoc*. We will show in the following sections that, as a way of balance, and in light of our results, it would appear that a tree is a good compromise between *ad hoc *and completely rigorous usage of the DAG.

We apply a minimal spanning tree (MST) algorithm to transform a DAG into a tree-structured hierarchy, by preserving the link between the child and the parent with the heaviest weight *w*, where *w *is the empirical conditional probability of having the child term given having the parent, based on a given PPI training set. Each GO term, in such a hierarchy, may still have more than one child term, but only one parent term (if the term itself is not the root of the hierarchy).

As a result of this transformation, there now exists a unique path from the root term to any non-root term. That is, let *G*_*d *_denote a term at the *d*-th level below the root. For example, *d *= 1 if the term is a child of the root. Then in our tree-structured hierarchy there is always a unique path of the form *G*_*d*_, *G*_*d*-1_, ..., *G*_1_, *G*_0_, with *G*_0 _being the root, and *G*_*i*-1 _being the parent of *G*_*i*_. For example, in Fig. 1(b), the result of applying our MST algorithm would be to drop the (*B*, *E*) link.

We propose to build a classifier in this setting based on the use of hierarchical conditional probabilities of the form $P(Y_{G_{d}}^{\left(i\right)}=1|X)$. Here *i *indexes a certain protein, and *G*_*d *_is a GO term of interest. The binary variable $Y_{G_{d}}^{\left(i\right)}$ = 1 indicates that protein *i *is labeled with *G*_*d*_; otherwise, it takes the value -1. Finally, X denotes the status of all of protein *i*'s neighbors in the PPI network, across all GO terms, as well as the status for protein *i *of all of the ancestor terms of *G*_*d*_. We will refer to X as the *neighborhood status *of *i*.

In the remainder of this section, we present certain model assumptions that in turn lead to a particular form for the probabilities $P(Y_{G_{d}}^{\left(i\right)}=1|X)$, as well as an efficient algorithm for their computation.

### Assumptions

We assume that labels on proteins obey a Markov property with respect to the PPI. That is, that the labeling of a protein is independent of any other proteins given that of its neighbors. Similarly, we assume that a Markov property holds on the GO tree-structured hierarchy, meaning that for a given protein the status of a GO term label is independent of that of the other terms, given that of its parent.

In addition, we assume that for any given protein *i*, the number of its neighbors labeled with a child term, among those labeled with the parent term, follows a binomial distribution, with probability depending on whether protein *i *is with the child or not. More precisely, we model

$$\begin{array}{ll} & P(k_{G_{ch}}|Y_{G_{ch}}^{\left(i\right)}=1,Y_{G_{pa}}^{\left(i\right)}=1;k_{G_{pa}})\\ = & B(k_{G_{ch}};k_{G_{pa}},p_{1})=\left(\begin{array}{c} k_{G_{pa}}\\ k_{G_{ch}} \end{array}\right)p_{1}^{k_{G_{ch}}}{\left(1-p_{1}\right)}^{k_{G_{pa}}-k_{G_{ch}}} \end{array}$$

and

$$\begin{array}{ll} & P(k_{G_{ch}}|Y_{G_{ch}}^{\left(i\right)}=-1,Y_{G_{pa}}^{\left(i\right)}=1;k_{G_{pa}})\\ = & B(k_{G_{ch}};k_{G_{pa}},p_{0})=\left(\begin{array}{c} k_{G_{pa}}\\ k_{G_{ch}} \end{array}\right)p_{0}^{k_{G_{ch}}}{\left(1-p_{0}\right)}^{k_{G_{pa}}-k_{G_{ch}}} \end{array}$$

where

• *G*_*ch *_is the child term; *G*_*pa *_is its parent;

• $k_{G_{ch}}$ is the number of *i*'s neighbors labeled with the *G*_*ch*_, and $k_{G_{pa}}$ is the number of neighbors labeled with *G*_*pa*_;

• *p*_1 _is the probability with which neighbors of *i *are independently labeled with *G*_*ch *_(being already labeled with *G*_*pa*_), given *i *is labeled with *G*_*ch*_;

• *p*_0 _is the probability with which neighbors of *i *are independently labeled with *G*_*ch *_(being already labeled with *G*_*pa*_), given *i *is NOT labeled with *G*_*ch *_but is labeled with *G*_*pa*_.

We refer to this overall set of model assumptions as the *Hierarchical Binomial-Neighborhood (HBN) *assumptions, in reference to their extension of the Binomial-Neighborhood (BN) assumptions of [4]. Note that the form of the probabilities above assumes that $k_{G_{ch}}$, the number of neighbors with the child term, is independent of the neighborhood size *N*, given $k_{G_{pa}}$, the number of neighbors with the parent. This condition seems reasonable since, recall that, by the *true-path rule*, only those among *i*'s neighbors that are labeled with the parent term can possibly have the child term. In other words, those neighbors with the child form a subset of those neighbors with the parent.

Parameters *p*_1 _and *p*_0 _are term-specific: different terms have different *p*_1 _and *p*_0_. For a given term *G*_*ch*_, all proteins share the same *p*_1 _and *p*_0_. They are estimated by pseudo-likelihood approach, from the labeled training data, separately for each *G*_*ch *_to be predicted. When calculating $k_{G_{ch}}$, $k_{G_{pa}}$, we use only the neighbors in the training set.

More specifically, assume there are *n *proteins in the training set, with *m *proteins labeled with *G*_*ch *_and *n *- *m *proteins not labeled with *G*_*ch*_. To simplify notation, let *k*_*ch*, *i *_and *k*_*pa*, *i *_be protein *i*'s training neighbors labeled with *G*_*ch *_and *G*_*pa*_, respectively. For the *m G*_*ch*_-annotated proteins, we have

*K*_*ch*, *i *_*~Binomial*(*k*_*pa*, *i*_, *p*_1_),

where $Y_{G_{ch}}^{\left(i\right)}$ = 1 and *i *= 1, 2, ..., *m*. With the Markov property assumption, the likelihood function for *p*_1 _based on all *G*_*ch*_-annotated proteins is

$$\begin{array}{ll} & L(p_{1}|k_{ch,1},...,k_{ch,n};k_{pa,1},...,k_{pa,n})\\ = & \Pi_{i=1}^{m}f(k_{ch,i}|k_{pa,i},p_{1})\\ = & \Pi_{i=1}^{m}\left(\begin{array}{c} k_{pa,i}\\ k_{ch,i} \end{array}\right)p_{1}^{k_{ch},i}{\left(1-p_{1}\right)}^{k_{pa,i}-k_{ch,i}}. \end{array}$$

The estimator for *p*_1 _is based on all *G*_*ch*_-annotated proteins' neighborhoods in the training set, and is the ratio of the total number of their *G*_*ch*_-annotated neighbors and the total number of their *G*_*pa*_-annotated neighbors, i.e.,

$${\hat{p}}_{1}=\frac{\sum_{i=1}^{m}k_{ch,i}}{\sum_{i=1}^{m}k_{pa,i}},$$

with $Y_{G_{ch}}^{\left(i\right)}$ = 1.

Similarly, the estimator for *p*_0 _is based on all *G*_*ch*_-unannoated proteins' neighborhoods in the training set, and is the ratio of the total number of their *G*_*ch*_-annotated neighbors and the total number of their *G*_*pa*_-annotated neighbors,

$${\hat{p}}_{0}=\frac{\sum_{j=1}^{n-m}k_{ch,j}}{\sum_{j=1}^{m}k_{pa,j}},$$

with $Y_{G_{ch}}^{\left(i\right)}$ = -1. Estimators ${\hat{p}}_{1}$ and ${\hat{p}}_{0}$ are formally pseudo-likelihood estimators.

An issue of estimation is the lack of data. Few data will affect the predictability and interpretability of the terms. Thus, we focus on terms with at least 5 proteins annotated with in the GO dataset. In principle, more formal work could be done, by using smoothing techniques and Empirical Bayes approaches, which we are exploring in our current work. It appears that improvement is not uniform, and the issue clearly requires separate consideration and will likely form a substantial component of a separate paper. Its subtlety likely is due to the well-known issue of classifiers doing well for classification while still being off-target for estimation [19].

Also notice that we use one-hop neighborhoods in this paper, i.e., neighbors that are directly connected to the protein of study. The extension to larger neighborhoods could be easily done, and would likely yield some improvement in predictive performance, but at the expense of some additional mathematical overhead, replacing the BN framework with one like those in [20-24]. Our choice to use a one-hop neighborhood structure here simply reflects a desire of maintaining a certain transparency in our model development, so as to emphasize primarily the effect of adding hierarchical information.

### Local Hierarchical Conditional Probability

By the Markov property assumed on the GO hierarchy, for any non-root term, only the parent affects its labelling. Therefore, to derive an expression for our hierarchical conditional probabilities $P(Y_{G_{d}}^{\left(i\right)}=1|X)$, we first concentrate on an expression for local hierarchical conditional probabilities of the form

(1) $$\begin{array}{ll} & P(Y_{G_{ch}}^{\left(i\right)}=1|Y_{G_{pa}}^{\left(i\right)}=1;X_{LOCAL})\\ = & P(Y_{G_{ch}}^{\left(i\right)}=1|Y_{G_{pa}}^{\left(i\right)}=1;k_{G_{ch}},k_{G_{pa}}). \end{array}$$

Applying Bayes' rule, we have

$$\begin{array}{ll} & P(Y_{G_{ch}}^{\left(i\right)}=1|Y_{G_{pa}}^{\left(i\right)}=1;k_{G_{ch}},k_{G_{pa}})\\ = & P(k_{G_{ch}},k_{G_{pa}}|Y_{G_{ch}}^{\left(i\right)}=1,Y_{G_{pa}}^{\left(i\right)}=1)\\ & \times P(Y_{G_{ch}}^{\left(i\right)}=1|Y_{G_{pa}}^{\left(i\right)}=1)/P(k_{G_{ch}},k_{G_{pa}}|Y_{G_{pa}}^{\left(i\right)}=1). \end{array}$$

For the first term in the numerator,

$$\begin{array}{ll} & P(k_{G_{ch}},k_{G_{pa}}|Y_{G_{ch}}^{\left(i\right)}=1,Y_{G_{pa}}^{\left(i\right)}=1)\\ = & P(k_{G_{ch}}|Y_{G_{ch}}^{\left(i\right)}=1,Y_{G_{pa}}^{\left(i\right)}=1;k_{G_{pa}})\\ & \times P(k_{G_{pa}}|Y_{G_{pa}}^{\left(i\right)}=1)\\ = & B(k_{G_{ch}};k_{G_{pa}},p_{1})\times P(k_{G_{pa}}|Y_{G_{pa}}^{\left(i\right)}=1); \end{array}$$

For the second term in the numerator, we use the plug-in estimate *f*, where *f *is defined to be the empirical probability of having the child term, given its having the parent, i.e.,

$$f=\hat{P}(Y_{G_{ch}}^{\left(i\right)}=1|Y_{G_{pa}}^{\left(i\right)}=1).$$

For the denominator, we apply the law of total probability and as a result, together with the two results above, the probability in (1) can be expressed as

(2) $$\begin{array}{ll} & P(Y_{G_{ch}}^{\left(i\right)}=1|Y_{G_{pa}}^{\left(i\right)}=1;k_{G_{ch}},k_{G_{pa}})\\ = & \frac{B(k_{G_{ch}};k_{G_{pa}},p_{1})\times f}{B(k_{G_{ch}};k_{G_{pa}},p_{1})\times f+B(k_{G_{ch}};k_{G_{pa}},p_{0})\times \bar{f}} \end{array}$$

where $\bar{f}$ = 1 - *f*.

### Global Hierarchical Conditional Probability

Equipped with the local hierarchical conditional probability, for any non-root GO term *G*_*d *_in the hierarchy, we now derive an expression for $P(Y_{G_{d}}^{\left(i\right)}=1|X)$, the probability that protein *i *is annotated with *G*_*d *_given its neighborhood status.

Note that by the *true-path rule *we have $P(Y_{G_{d}}^{\left(i\right)}=1,Y_{G_{d-1}}^{\left(i\right)}=-1|X)=0$, where *G*_*d*-1 _is the parent of *G*_*d*_.

Hence,

(3) $$\begin{array}{ll} & P(Y_{G_{d}}^{\left(i\right)}=1|X)\\ = & P(Y_{G_{d}}^{\left(i\right)}=1,Y_{G_{d-1}}^{\left(i\right)}=1|X).\\ = & P(Y_{G_{d}}^{\left(i\right)}=1|Y_{G_{d-1}}^{\left(i\right)}=1;X)\times P(Y_{G_{d-1}}^{\left(i\right)}=1|X). \end{array}$$

This logic easily extends recursively back through all ancestors of *G*_*d*_, and thus the conditional probability (3) can be factorized as

$$\begin{array}{ll} & P(Y_{G_{d}}^{\left(i\right)}=1|X)\\ = & \Pi_{m=1}^{d}P(Y_{G_{m}}^{\left(i\right)}=1|Y_{G_{m-1}}^{\left(i\right)}=1;X_{LOCAL_{m}})\\ = & \Pi_{m=1}^{d}P(Y_{G_{m}}^{\left(i\right)}=1|Y_{G_{m-1}}^{\left(i\right)}=1;k_{G_{m}},k_{G_{m-1}}), \end{array}$$

where $X_{LOCAL_{m}}$ is the local hierarchical neighborhood information on the parent-child GO term pair, *G*_*m *_and *G*_*m*-1_.

Importantly, note that due to the form of the factorization, the global conditional probability for *G*_*d *_is no greater than that for its parent *G*_*d*-1_, i.e., we have the inequality

$$\begin{array}{ll} & P(Y_{G_{d}}^{\left(i\right)}=1|X)\\ = & \Pi_{m=1}^{d}P(Y_{G_{m}}^{\left(i\right)}=1|Y_{G_{m-1}}^{\left(i\right)}=1;X_{LOCAL_{m}})\\ \leq & \Pi_{m=1}^{d-1}P(Y_{G_{m}}^{\left(i\right)}=1|Y_{G_{m-1}}^{\left(i\right)}=1;X_{LOCAL_{m}})\\ = & P(Y_{G_{d-1}}^{\left(i\right)}=1|X). \end{array}$$

As we go down along the path from the root in the hierarchy, the probability that protein *i *is labeled with a more specific term is always no more than the probability of any of its ancestors. If the label of a term is predicted as -1, according to some pre-chosen threshold, the labels for every descendent below will also be assigned as -1. Thus, our model is guaranteed to produce GO term label assignments that comply with the *true-path rule*. Most existing methods for protein function prediction use ad-hoc enforcement to correct predictions in order to maintain *true-path *consistency.

### Algorithm

Classification using our Hierarchical Binomial-Neighborhood (HBN) model may be accomplished using a straightforward top-to-bottom algorithm. Specifically, for a given protein *i*, and a pre-determined threshold *t*, we proceed from the child terms of the root in the MST representation of the GO hierarchy in the following fashion.

**initialize **PROB = 1

**for ***m *= 1: *d*_*max*_,

      **while ∃ **unlabeled terms *G*_*m *_at level *m*,

      **compute **${\text{PROB}}_{G_{m}}\leftarrow {\text{PROB}}_{G_{m-1}}\times$

         $P(Y_{G_{m}}^{\left(i\right)}=1|Y_{G_{m-1}}^{\left(i\right)}=1;X_{LOCAL_{m}})$

      **if **${\text{PROB}}_{G_{m}}$ >*t*, set $Y_{G_{m}}^{\left(i\right)}$ = 1

      **else **set $Y_{G_{m}}^{\left(i\right)}$ = -1 and propagate to all descendants of *G*_*m*_

   **end**

end

Notice that setting the labels at each step is not necessary. However, doing so facilitates the computation efficiency, by avoiding the calculation of the probabilities below the threshold. By letting *t *= 0, we can obtain all probabilities. The fact that we can do this is a direct outcome of the fact that our predictions are guaranteed to obey *the true-path rule*.

For a given protein, the algorithm requires at most *O*(*N*_*GO*_) steps, where *N*_*GO *_is the number of GO terms, and therefore, for *N*_*Protein *_proteins, no more than *O*(*N*_*Protein*_*N*_*GO*_) steps are needed. Hence, the algorithm is linear in the size of both the PPI and the GO networks. In practice, it has been found to be quite fast, particularly because each protein can be expected to have a large proportion of -1 labels, and once a -1 is assigned to a term it is simply propagated to all descendant terms.

## Results

### Data

The PPI data used in this paper is from the yeast *Saccharomyces cerevisiae*, as updated in January 2007 at . There are 5143 genes (nodes) and 31190 non-redundant physical interactions (edges), after deleting self-interacting and unannotated nodes, and genetic interactions.

The Gene Ontology used is *biological process*, updated in June 2006, as posted at . From the biological perspective, more specific terms are more interesting than less specific ones, and we therefore only predict for terms with 300 or less genes annotated in the database. As a result, the entire *biological process *ontology breaks down into 47 sub-hierarchies. In addition, to avoid extremes with little to no information, we only predict for terms with at least 5 genes. We also delete GO:0000004, biological function unknown. The total number of terms predicted is 1037. The GO term annotations used to train the model are updated in June 2006, from .

From the initial data, a set of labels is constructed in a way that follows the *true-path rule*. Specifically, for any protein-term association in the data, we assign a +1 label to the term for that protein, as well as to all of the ancestors in the transitive closure of that term in the GO hierarchy. We repeat this for all protein-term associations to get the set of all positive labels. We assign -1 to all other protein-term pairs.

Please visit  for the datasets used in this paper and the Matlab scripts for the HBN algorithm.

### Cross-Validation Study

We apply our Hierarchical Binomial-Neighborhood (HBN) method, as well as the "Nearest-Neighbor" (NN) algorithm and the Binomial-Neighborhood (BN) method of [4], to the data just described, using a 5-fold cross-validation design. The HBN and BN methods each produce a probability of protein-term association, while the NN algorithm similarly produces a number between 0 and 1 (i.e., the fraction of a protein's neighbors in the PPI network possessing the term in question). For each test fold, representing 20% of the proteins, all GO term annotations are taken as unknown, and predictions of protein-term associations are made with each of the three methods, based on comparison of their output to a threshold *t *∈ [0, 1], using the annotations in the other four folds as training data.

### Evaluation

We use three metrics by which to evaluate the performance characteristics of each classification method. The first is the standard Receiver Operating Characteristic (ROC) curve, which evaluates a classifier's performance in a manner that aggregates across all terms. We examine ROC curves both for the overall GO hierarchy and within each of the 47 sub-hierarchies.

Since the ROC curve, as a metric, is 'flat', in that it ignores any hierarchical structure among terms, we use as a second metric a hierarchical performance measure, called *hF_β_*, proposed in [25,26] and defined as follows. For a hierarchy of GO terms and any protein *i *that is annotated with the hierarchy root, first take the transitive closure of all of the most specific +1 predictions and change -1's into +1's, if there is any. Note that this step is only necessary here for "Nearest-Neighbor" and the "Binomial-Neighborhood" method.

Next, for each protein *i*, calculate the true positive (TP), false positive (FP), and false negative (FN) counts, based on the true labels of all terms in the hierarchy and the corrected predictions, denoted as *TP*_*i*_,

*FP*_*i *_and *FN*_*i*_, respectively. Define hierarchical precision (*hP*) and hierarchical recall (*hR*) as

$$\begin{array}{cc} hP=\frac{\sum_{i=1}^{\#proteins}TP_{i}}{\sum_{i=1}^{\#proteins}TP_{i}+FP_{i}}, & hR\frac{\sum_{i=1}^{\#proteins}TP_{i}}{\sum_{i=1}^{\#proteins}TP_{i}+FN_{i}}. \end{array}$$

The value *hF_β _*is then defined as a weighted combination of *hP *and *hR*, in the form

$$hF_{\beta }=\frac{(\beta^{2}+1)hP\times hR}{\beta^{2}hP+hR},$$

where *β *∈ [0, ∞) is a tuning parameter. In this paper, we use *hF*_1 _with equal weights on precision and recall, simply denoted as *hF*. Note that *hF*, *hP *and *hR *are all scaled between 0 and 1, with higher *hF *indicating better performance over the hierarchy.

Lastly, because accurate positive predictions are of most biological interest in this area, and because predictions of terms increasingly deeper in the GO hierarchy are of increasingly greater use, we examine the positive predictive value (PPV) of each of the methods, as a function of depth in the hierarchy. However, as the prevalence of known terms tends to decrease substantially with depth, and PPV decreases similarly with decreasing prevalence, we normalize PPV by prevalence to allow meaningful comparison across depths. Specifically, we compute a log-odds version of PPV in the form

(4) $$\text{LO-PPV}=\log\frac{PPV/(1-PPV)}{f/(1-f)},$$

where *f *is the prevalence of a given term. This quantity therefore indicates relative performance of a given classifier, in comparison with a method that simply predicts proteins to have a given term with *a priori *probability *f*.

### An Illustration

**Figure 3:**
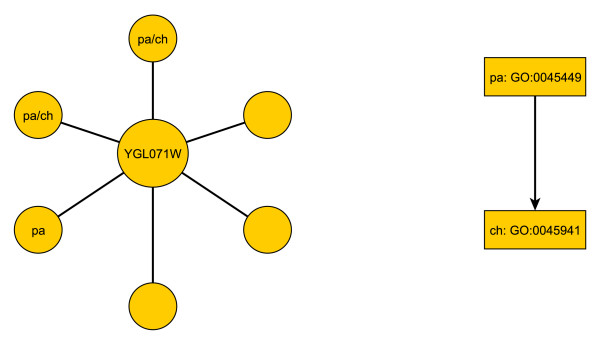
**Illustration of HBN's working mechanism**. The plot shows (a) protein YGL017W and its neighborhood, (b) Small GO hierarchy. Three neighbors are labeled with the parent term GO:0045449; two of them are labeled with the child term GO:0045941. We want to predict whether YGL017W is labeled with GO:0045941.

To better appreciate the performance gains from HBN that we describe momentarily below, we first present an illustrative example. Consider protein YGL017W (AFT1) and its neighborhood, as depicted in Fig. 3(a). Knowing that YGL017W is labeled with the parent term *G*_*pa *_= GO:0045449, or *regulation of transcription*, we want to predict whether YGL017W is labeled with the child term *G*_*ch *_= GO:0045941, or *positive regulation of transcription*. All six neighbors are in the training set, and used together with other training nodes to estimate parameters. Three out of six neighbors are labeled with *G*_*pa*_, and two with *G*_*ch*_. The prediction from HBN results from applying a threshold to Equation. The analogous probability for BN is given by

$$P(Y_{G}=1|k,N)=\frac{B(k;N,p_{1}^{*})\times f^{*}}{B(k;N,p_{1}^{*})\times f^{*}+B(k;N,p_{0}^{*})\times {\bar{f}}^{*}},$$

where

• *G *is the target GO term, GO:0045941;

• *k *is the number of training neighbors labeled with *G*;

• *N *is the training neighborhood size;

• $p_{1}^{*}$ is the probability with which neighbors are independently labeled with *G*, given protein YGL017W is labeled with *G*;

• $p_{0}^{*}$ is the probability with which neighbors are independently labeled with *G*, given protein YGL017W is NOT labeled with *G*;

• f* is the relative frequency of *G *in the training set, and ${\bar{f}}^{*}$ = 1 - f*.

Table 1 contains the parameters for each of the three classification methods, and the output they produce. HBN provides substantially more evidence for YGL017W being labeled with GO term GO:0045941, which is in fact the case. With a threshold *t *= 0.5, only HBN provides a correct positive prediction. The improvement here comes from the additional information provided by including parent-term information.

**Table 1:** Parameters from Nearest-Neighbor (NN), Binomial-Neighborhood (BN) and Hierarchical Binomial-Neighborhood (HBN)

NN	BN	HBN
*k *= 2	*k *= 2	*k *= 2
*N *= 6	*N *= 6	*N *= 6
.	$p_{1}^{*}$ = 0.0661	*p*_1 _= 0.2927
.	$p_{0}^{*}$ = 0.0085	*p*_0 _= 0.0992
.	f* = 0.0106	*f *= 0.2186
*P *= 0.3333	*P *= 0.3381	*P *= 0.6566

This table contains the parameters and the corresponding probabilities estimated by the three methods, as discussed in the paper, when predicting whether yeast gene YGL017W has GO term GO:0045941, *positive regulation of transcription*.

### Cross-Validation Results

**Figure 4:**
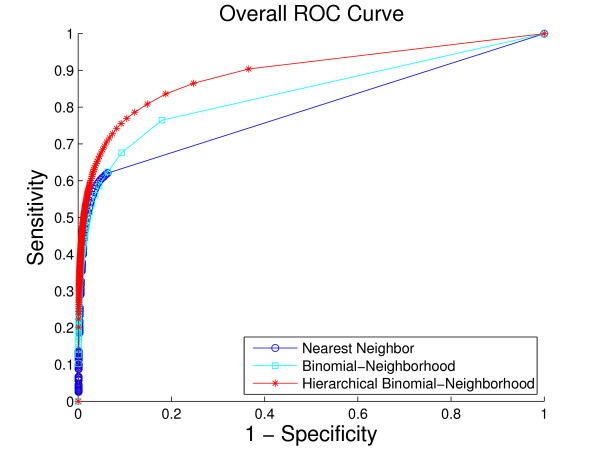
**Overall method performance comparison by ROC curve**. This plot demonstrates the ROC curves of the three methods based on the 5-fold cross-validation study on the whole yeast genome. Colors: HBN (red); BN (light blue); NN (blue).

**Figure 5:**
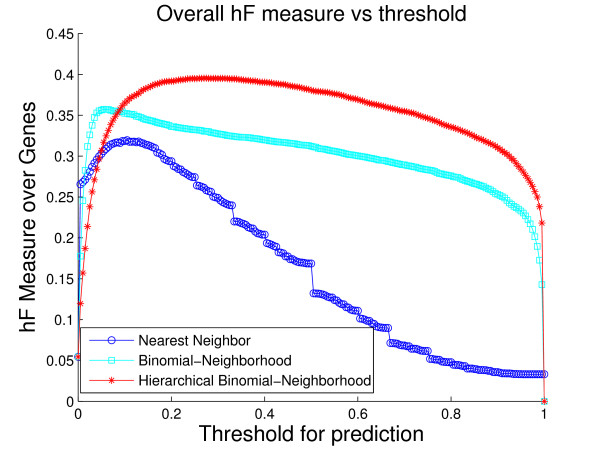
**Overall method performance comparison by *hF *measure**. This plot demonstrates the curves of *hF *measure of the three methods against predicting threshold, based on the 5-fold cross-validation study on the whole yeast genome. Colors: HBN (red); BN (light blue); NN (blue).

**Figure 6:**
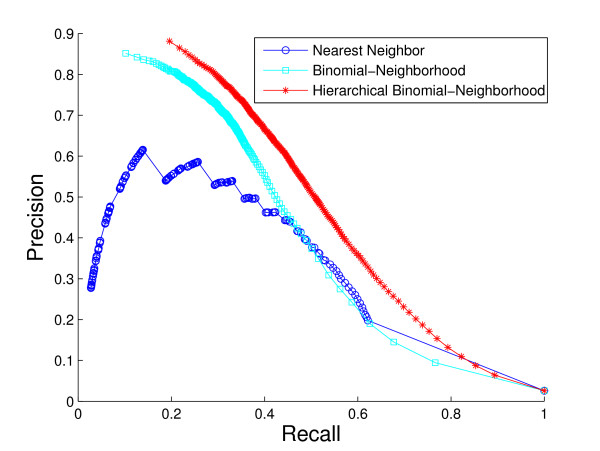
**Overall method performance comparison by precision and recall**. This plot demonstrates the precision versus recall curves of the three methods based on the 5-fold cross-validation study on the whole yeast genome. Colors: HBN (red); BN (light blue); NN (blue).

A comparison of the overall performance of the three methods, by ROC curves and the *hF *measure, is shown in Fig. 4 and Fig. 5, respectively. We are also interested in visualizing precision versus recall, shown in Fig. 6. A total of 1037 GO terms are studied on 5143 proteins. Sensitivity, specificity and *hF *are calculated by combining, within each of the 5 folds, the true positive (TP), false positive (FP), true negative (TN) and false negative (FN) counts, over all proteins and all terms for varying thresholds, and averaging across folds. Precision and recall are defined as $\text{precision}=\frac{TP}{TP+FP}$, $\text{recall}=\frac{TP}{TP+FN}$. The HBN method outperforms the other two methods by a clear margin in all figures, except at very small thresholds (*t *< 0.1) in the *hF *plot. Comparison of the area under the curve (AUC) for each method, in the ROC and *hF *plots, through a simple paired *t*-test on four degrees of freedom, confirms this observation, i.e., *p *< 10^-5 ^for comparison of HBN with BN and with NN. The gains of HBN over BN directly reflects the benefit of effectively integrating the GO hierarchical information into the construction of our classifier.

Recall that, as a result of our predicting only for GO terms annotated with less than 300 proteins in the database, the full *biological process *hierarchy actually breaks into 47 sub-hierarchies. Examination of performance on these sub-hierarchies provides some sense of the extent to which the HBN performance improvements are uniform across the GO hierarchy. We compute a ROC curve and *hF *plot for each of the sub-hierarchies (See additional file 1: ROC curves and *hF *plots for 47 sub-hierarchies in cross-validation study). Numerical comparison of the corresponding AUCs finds, at a 5% significance level that HBN improves on BN in 38 of the 47 sub-hierarchies, according to the ROC curves, 19 of the sub-hierarchies, according to the *hF *plots, and 18 commonly between them. Conversely, BN outperforms HBN in only 1 of the 47 sub-hierarchies, according to the ROC curves, and 9 of the sub-hierarchies, according to the *hF *plots. (NN was uniformly the worse performer.)

These ROC plots are constructed using the original BN (and NN) predictions, without any correction for "true-path" consistency. However, the overwhelming improvement of HBN over BN indicated by the ROC curves is actually similar when the initial predictions of BN are post-processed by applying transitive closure. Specifically, HBN improves on BN in 28 of the sub-hierarchies, while BN outperforms HBN in only 4 sub-hierarchies. These results strongly suggest the validity of our premise as to the importance of incorporating hierarchical information in the GO database in the initial construction of a classifier. The *hF *plots, which incorporate transitive closure for BN (and NN) directly into their definition, and are designed to provide a more accurate summary of classification accuracy with hierarchically related class labels, support this conclusion. The gains of HBN over BN, although reduced, are still substantial, with HBN outperforming BN in just over 40% of the 47 hierarchies, and BN outperforming HBN, in less than 20%.

**Figure 7:**
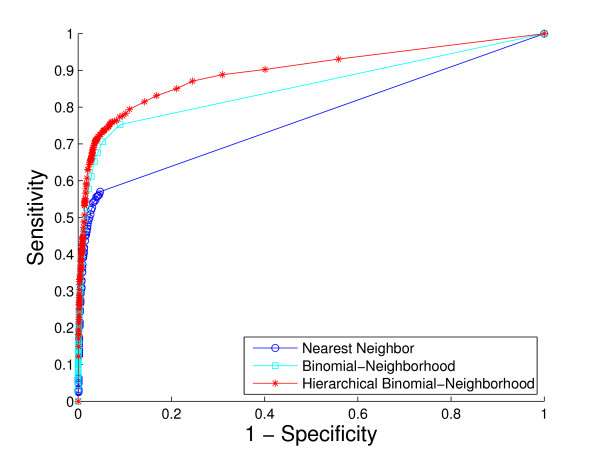
**Method performance comparison by ROC curve on sub-hierarchy GO:0050896**. The plot shows the ROC curves of the three methods based on the 5-fold cross-validation study on the sub-hierarchy with root GO term GO:0050896, *response to stimulus*. Colors: HBN (red); BN (light blue); NN (blue).

**Figure 8:**
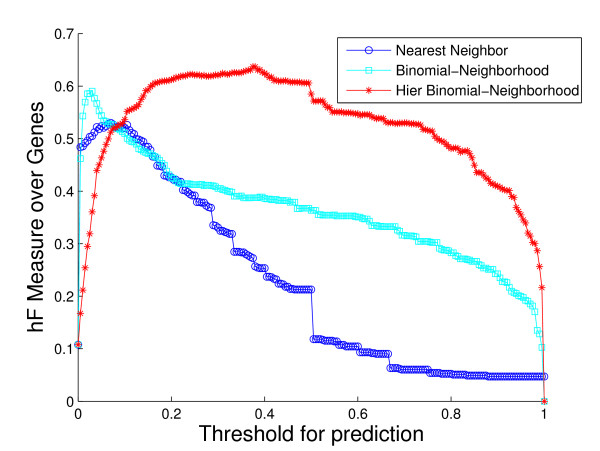
**Method performance comparison by *hF *on sub-hierarchy GO:0050896**. The plot shows the curves of *hF *measure of the three methods against predicting threshold, based on the 5-fold cross-validation study on the sub-hierarchy with root GO term GO:0050896, *response to stimulus*. Colors: HBN (red); BN (light blue); NN (blue).

As an illustration, consider the performance on the sub-hierarchy corresponding to Fig. 7 and Fig. 8. The root term of the sub-hierarchy is GO:0050896, *response to stimulus*, with 72 more specific terms below it, 40 out of which are predicted for 536 proteins annotated with root. The shapes and locations of the curves in these plots are similar to those in Fig. 4 and Fig. 5, with arguably a more substantial improvement from HBN in the *hF *plot. For instance, using a threshold of *t *= 0.5 for prediction, HBN produces an *hF *measure nearly 254% and 60% higher than NN and BN, respectively (*hF*_*NN *_= 0.16, *hF*_*BN *_= 0.35, and *hF*_*HBN *_= 0.56).

**Figure 9:**
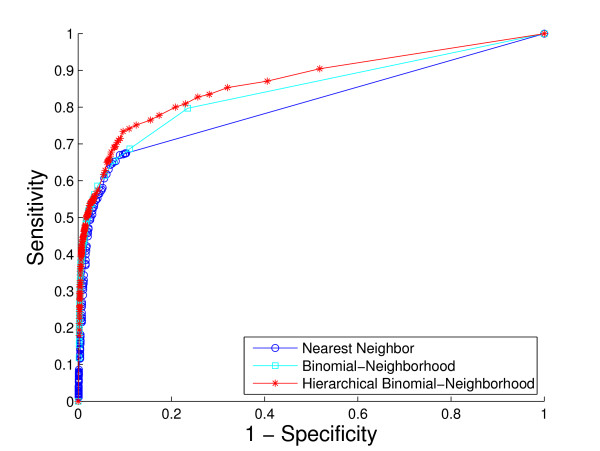
**Method performance comparison by ROC curve on sub-hierarchy GO:0019538**. The plot shows the ROC curves of the three methods based on the 5-fold cross-validation study on the sub-hierarchy with root GO term GO:0019538, *protein metabolism*. Colors: HBN (red); BN (light blue); NN (blue).

**Figure 10:**
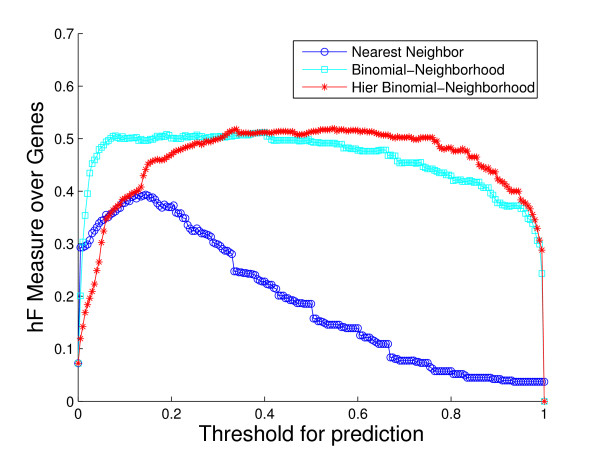
**Comparison of method performance by *hF *on sub-hierarchy GO:0019538**. The plot shows the curves of *hF *measure of the three methods against predicting threshold, based on the 5-fold cross-validation study on the sub-hierarchy with root GO term GO:0019538, *protein metabolism*. Colors: HBN (red); BN (light blue); NN (blue).

In contrast, Fig. 9 and Fig. 10 show an example of a sub-hierarchy in which the performance of HBN and BN are too close to declare one or the other better. This sub-hierarchy has root term GO:0019538, *protein metabolism*. Examination of the predictions seems to suggest that the comparatively poorer relative performance of HBN in this sub-hierarchy is due to its over-optimistic positive predictions, i.e., HBN produces a higher rate of false positives (FP) that lowers the hierarchical precision (*hP*) and hence the *hF *measure.

**Figure 11:**
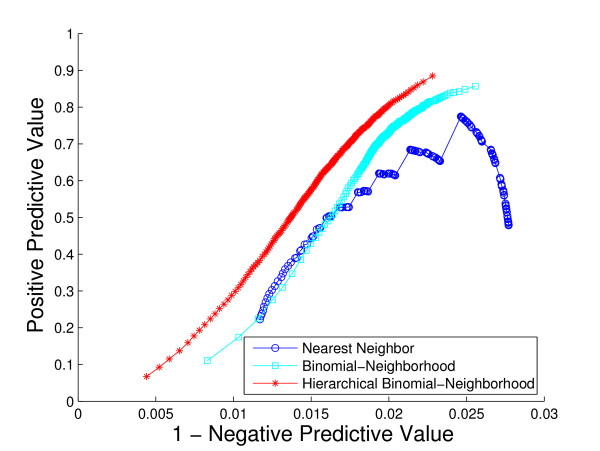
**Visualization of the averaged positive predictive value comparison**. The plot contains the curves of the averaged positive predictive values (PPV) over cross-validation folds of the three methods, against 1-NPV, the averaged negative predictive value (NPV). Colors: HBN (red); BN (light blue); NN (blue).

**Figure 12:**
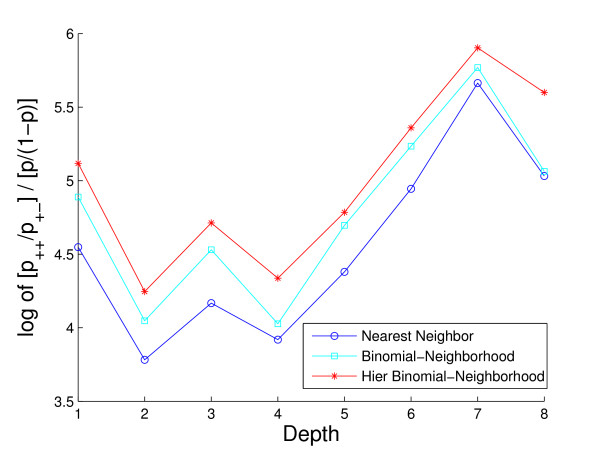
**Visualization of the averaged log-odds positive predictive value comparison on GO hierarchy depth**. The plot demonstrates the curves of the averaged log-odds PPV over cross-validation folds of the three methods for NPV = 0.987, as a function of the GO hierarchy depth. Colors: HBN (red); BN (light blue); NN (blue).

Lastly, Fig. 11 and Fig. 12 contain plots summarizing the positive predictive value (PPV) of the three methods. In Fig. 11, we show how the averaged PPV varies against the averaged negative predictive value (NPV), over all proteins and GO terms for which all three methods produced at least one positive prediction, averaged over the five folds (PPV versus 1-NPV). We see that the HBN method has consistently higher PPV across all values of NPV. At an NPV of 0.987 (i.e., 1 - NPV = 0.013), for example, where the PPV for HBN is nearly 50% (i.e., PPV = 0.465), that for BN and NN are only roughly 30% (i.e., PPV = 0.310 and 0.326, respectively). That is, for the same rate of correct negative predictions, HBN produces nearly one in two correct positive predictions, while the other two methods produce not quite one in three. Note that the extremely high NPV values for all three methods are largely a result of the similarly high prevalence of -1 labels in the database.

Shown in Fig. 12 is the log-odds PPV of all three methods, for NPV = 0.987, as a function of depth in the GO hierarchy. We see that the improvement in positive predictive capabilities of HBN is fairly uniform across depths. A one-sided paired *t*-test at each depth confirmed the differences to be highly significant (i.e., *p*-values roughly 0.001 or less) at depths 3, 4, and 5, but not at depths 1, 2, 6, 7, or 8. We note, however, that the lack of significance at the latter depths is likely partly driven by sample size, since at each of these depths there were less than 30 cases of positive protein-term predictions by all three methods used in calculating LO-PPV, while at the other three depths there were well in excess of 100.

### In Silico Validation Results

Recall that the above results are based on gene-GO term annotations in the January 2007 GO database. As an *in silico *proxy to *in vitro *validation, beyond that of the cross-validation study, we examined the performance of HBN, in comparison to NN and BN, when applied to new gene-GO term annotations found in the updated May 2007 database. Here our goal is to evaluate the robustness of our cross-validation results for predicting naturally occurring unknowns (i.e., as opposed to those left out in a random fashion through cross-validation).

We applied HBN, BN, and NN in each of the 47 sub-hierarchies to genes that (i) were annotated with only the root term in the June 2006 database, and (ii) were assigned more specific functions in that sub-hierarchy in the May 2007 database. There were a total of 508 genes that had received at least one new annotation in one of the sub-hierarchies, with as few as 1 gene and as many as 74 genes per hierarchy. There were 33 sub-hierarchies having such genes. The methods were compared for their accuracy through the *hF *function. We present the *hF *plots for only those sub-hierarchies (17) with sufficiently many annotations to yield meaningful results (See additional file 2: *hF *plots for 17 sub-hierarchies in *in silico *study); the *hF *measures for the others are trivial, due to too few new annotations. Over 40% (i.e., 7 out of 17) of these *hF *plots find HBN to work best in correctly detecting more specific associations, over a reasonably broad range of threshold values; in the majority of the remaining plots, HBN yields results similar to the at least one of the two other methods.

**Figure 13:**
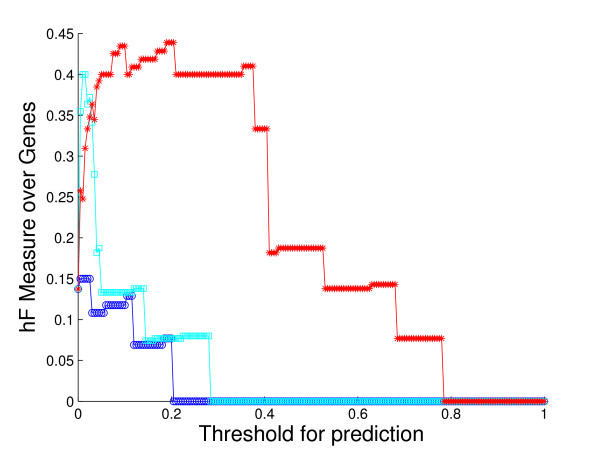
***hF *plots for new predictions on sub-hierarchy GO:0050896**. The plot shows the *hF *curves of the three methods based on the updated annotation for sub-hierarchy with root term GO:0050896, *response to stimulus*, as discussed in the *in silico *validation study. Colors: HBN (red); BN (light blue); NN (blue).

**Figure 14:**
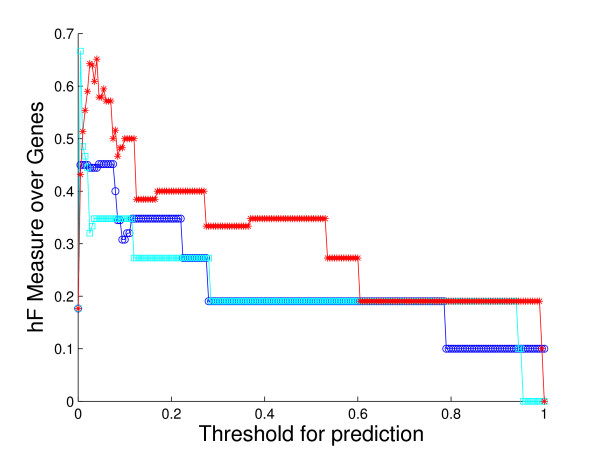
***hF *plots for new predictions on sub-hierarchy GO:0019538**. The plot shows the *hF *curves of the three methods based on the updated annotation for sub-hierarchy with root term GO:0019538, *protein metabolism*, as discussed in the *in silico *validation study. Colors: HBN (red); BN (light blue); NN (blue).

Overall, most of the plots are consistent with the cross-validation results. Interestingly, however, there are a number of cases where HBN clearly outperforms NN and BN by a larger margin in the *in silico *validation than in the cross-validation study. For example, for the sub-hierarchy with root term *response to stimulus*, the new *hF *curve for HBN exceeds that for BN by as much as 300%, dominating those for the other two methods for most of the thresholds. See Fig. 13. In addition, in some sub-hierarchies where HBN does not perform best in cross-validation, its *hF *curve is significantly improved in the *in silico *study, and in fact outperforms the other two methods. The sub-hierarchy with root term *protein metabolism *is of this sort. The *hF *curve for HBN in Fig. 14 dominates the other two methods for almost 60% of the possible threshold values on the new predictions, even though HBN works no better than BN in the cross-validation study.

Overall, these results suggest that the performance advantages of HBN indicated by the cross-validation study are, if anything, potentially understated.

## Discussion

For a well-studied organism, such as *S. cerevisiae*, one can make certain inferences about genes for which there is no annotated function. First, it is likely that the gene has low sequence similarity to any gene of known function, thus preventing the most straightforward computational methods of predicting gene function. Secondly, it is likely that no altered phenotype is observed upon protein overexpression, knockdown, or knockout, foiling first-pass experimental attempts to discover gene function. In these cases, the next step would involve more elaborate experimental methods, which would typically be guided by a co-expression analysis of publicly available microarray data. The experiments selected will, in general, be time-consuming, costly, different for each gene being investigated, and offer modest chances for success. Thus, the development of more sophisticated and accurate methods of computational prediction of function which could precisely guide experimental activity remains a top priority.

Biological and biomedical ontologies have become a prominent, and perhaps indispensable, tool in bioinformatics and biological research. GO in particular has been used in numerous papers to detect biological process enrichment of co-expressed genes, identify biological processes associated with disease, etc. However in the vast majority of applications the hierarchical nature of GO is actually not being used directly. For example, in enrichment testing such as GSEA or GNEA we typically test for every biological process if the differentially expressed genes in some condition are associated with this process more than expected by chance.

Thus while GO and other ontologies obviously organize biological knowledge in an intuitive fashion, the structure is not typically exploited for actual inference by predictive analysis tools. This is rather different from evolutionary analysis tools and genetics frameworks where probabilistic ancestor/descendant relationships in phylogenies (hierarchies) are exploited very directly with substantial practical and theoretical benefits.

Our work here suggests that similar developments of probabilistic frameworks are not only feasible, but promising, for improved protein function inference with gene ontologies. In addition, it suggests the need for further research to be done to clarify the utility of different representations for such purposes. Finally, it also raises the prospect of re-engineering ontologies or other similar representations, from the perspective of seeking to provide maximal value for probabilistic inference programs.

## Conclusion

We have developed a probabilistic framework for automated prediction of protein function using relational information (e.g., a network of protein-protein interactions) which exploits the hierarchical structure of ontologies, and guarantees the predictions obey a 'true-path' annotation rule. We have evaluated the performance of our method and compared it with two other network-based methods by both cross-validation and an *in silico *study, on the genome of yeast, for terms from the biological process category in the Gene Ontology. Results showed that our proposed method, by utilizing the ontological structure, significantly improved the prediction accuracy and the capability of detecting positive annotations over the hierarchies. Furthermore, our analysis suggests that such improvement persists across the ontology depths.

## Authors' contributions

XJ carried out the statistical study, implemented and performed the computation, drafted the manuscript. NN prepared the datasets and helped the computation. MS interpreted the results and took part in the analysis. SK participated in the design of the study and the analysis. EDK conceived of the study, participated in its design, supervised the analysis and finalized the manuscript. All authors read and approved the final manuscript.

## Supplementary Material

Additional file 1 **ROC curves and *hF *plots for 47 sub-hierarchies in cross-validation study**. This file contains the ROC curves and plots of *hF *score versus predicting threshold of the three methods for 47 individual sub-hierarchies in the 5-fold cross-validation study. The root term ID's and names of the root terms, the sizes of sub-hierarchies, numbers of terms and genes predicted within sub-hierarchy are also shown. Colors: HBN (red); BN (light blue); NN (blue). Click here for file

Additional file 2 ***hF *plots for 17 sub-hierarchies in *in silico *study**. This file contains the plots of *hF *score versus threshold of the three methods for individual sub-hierarchies in the *in silico *validation study. The root term ID's and names of the root terms, the sizes of sub-hierarchies, numbers of terms and genes predicted within sub-hierarchy are also shown. Colors: HBN (red); BN (light blue); NN (blue). Click here for file
