# Supplementary material for: Integration of relational and hierarchical network information for protein function prediction
Source: BMC Bioinformatics. 2008 Aug 22;9:350. doi: 10.1186/1471-2105-9-350 (PMC2535605; doi:10.1186/1471-2105-9-350)
Supplement: Additional file 1 — ROC curves and hF plots for 47 sub-hierarchies in cross-validation study. This file contains the ROC curves and plots of hF score versus predicting threshold of the three methods for 47 individual sub-hierarchies in the 5-fold cross-validation study. The root term ID's and names of the root terms, the sizes of sub-hierarchies, numbers of terms and genes predicted within sub-hierarchy are also shown. Colors: HBN (red); BN (light blue); NN (blue). [file 1471-2105-9-350-S1.pdf]

Integration of Relational and Hierarchical Network Information for Protein Function Prediction

Additional file - 1

Xiaoyu Jiang, Naoki Nariai, Martin Steffen, Simon Kasif, Eric D. Kolaczyk

---

1. **Hierarchy root: GO:0006139, nucleobase, nucleoside, nucleotide and nucleic acid metabolism**
   Number of terms in hierarchy = 154
   Number of terms predicted = 59
   Number of genes predicted = 1327

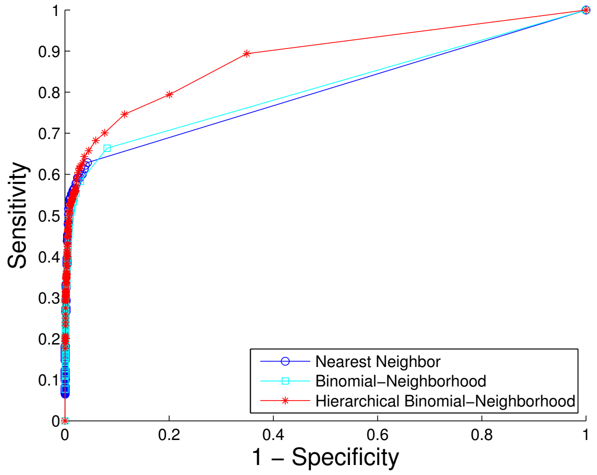

Figure 1: ROC curves by 5-fold cross-validation study.

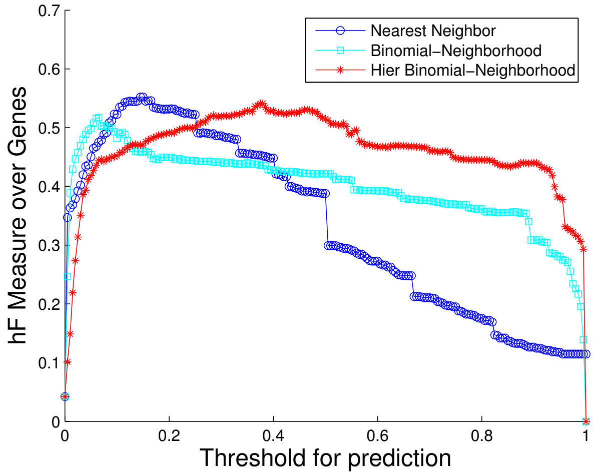

Figure 2: hF measure v.s. threshold.

2. **Hierarchy root: GO:0006259, DNA metabolism**
Number of terms in hierarchy = 108
Number of terms predicted = 68
Number of genes predicted = 501

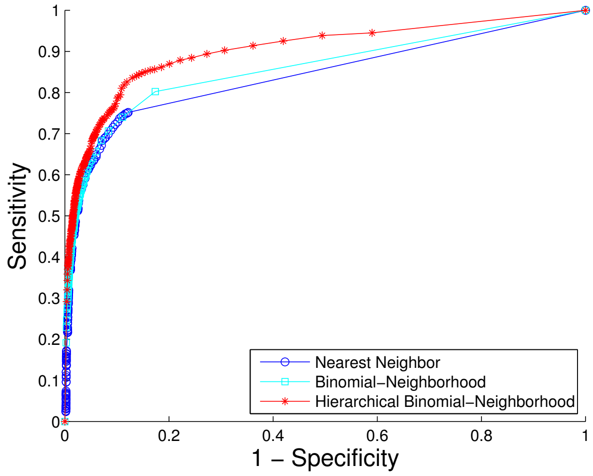

Figure 1: ROC curves by 5-fold cross-validation study.

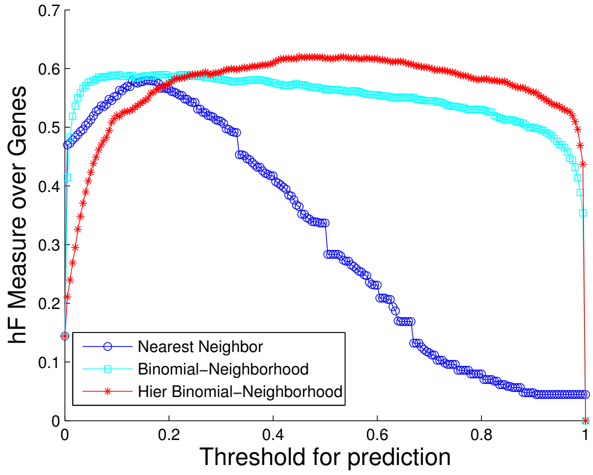

Figure 2: hF measure v.s. threshold.

3. **Hierarchy root: GO:0006351, transcription, DNA-dependent**
Number of terms in hierarchy = 57
Number of terms predicted = 37
Number of genes predicted = 426

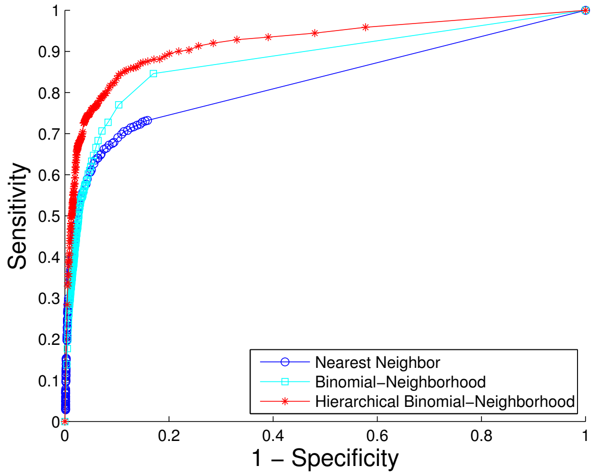

Figure 1: ROC curves by 5-fold cross-validation study.

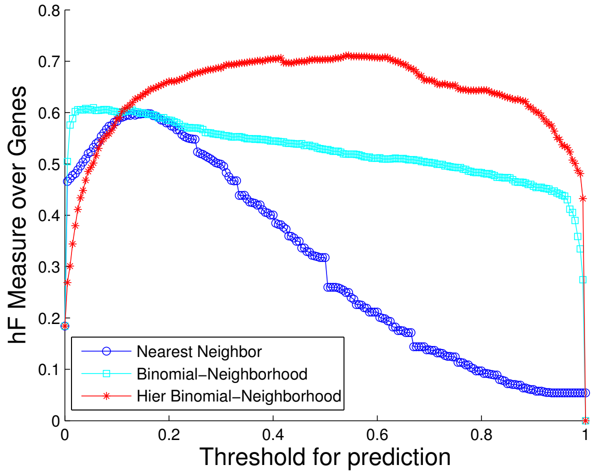

Figure 2: hF measure v.s. threshold.

4. **Hierarchy root: GO:0006396, RNA processing**
Number of terms in hierarchy = 57
Number of terms predicted = 24
Number of genes predicted = 322

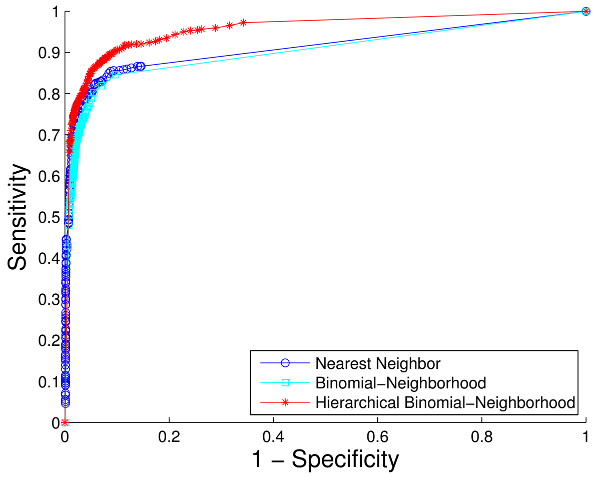

Figure 1: ROC curves by 5-fold cross-validation study.

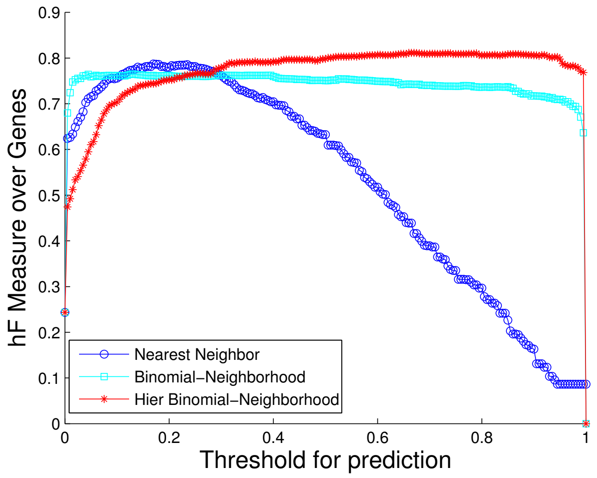

Figure 2: hF measure v.s. threshold.

5. **Hierarchy root: GO:0006412, protein biosymthesis**
Number of terms in hierarchy = 66
Number of terms predicted = 28
Number of genes predicted = 464

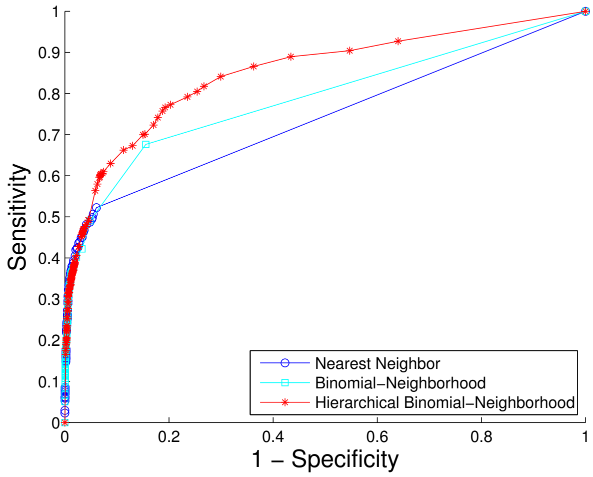

Figure 1: ROC curves by 5-fold cross-validation study.

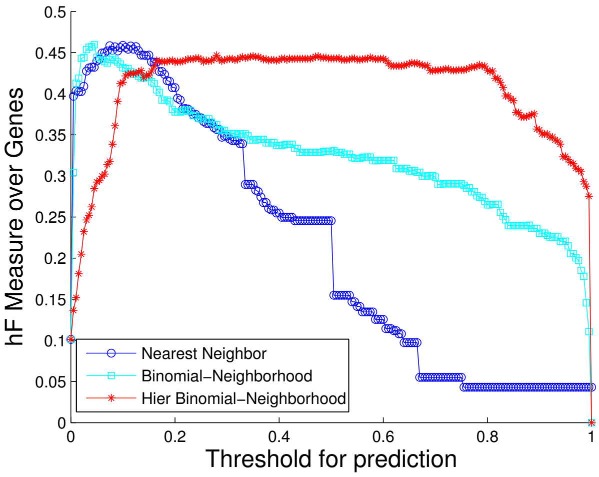

Figure 2: hF measure v.s. threshold.

6. **Hierarchy root: GO:0006464, protein modification**
Number of terms in hierarchy = 86
Number of terms predicted = 43
Number of genes predicted = 430

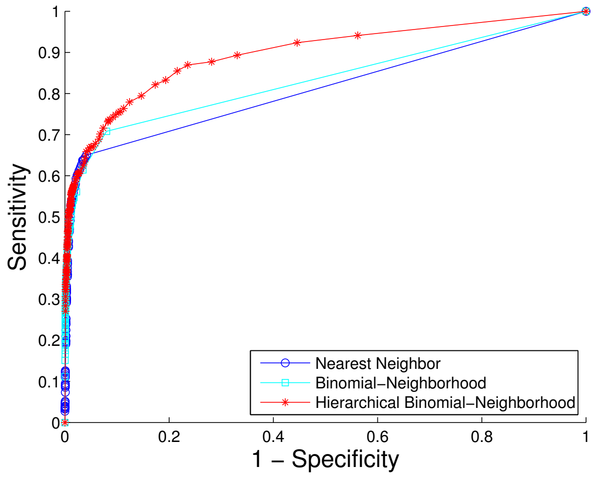

Figure 1: ROC curves by 5-fold cross-validation study.

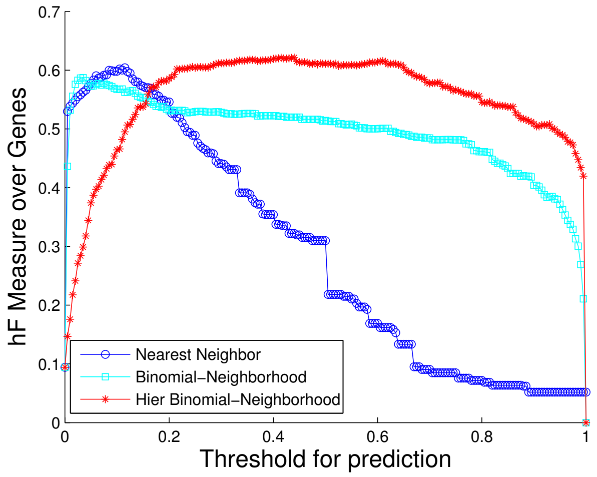

Figure 2: hF measure v.s. threshold.

7. **Hierarchy root: GO:0006810, transport**
Number of terms in hierarchy = 175
Number of terms predicted = 67
Number of genes predicted = 906

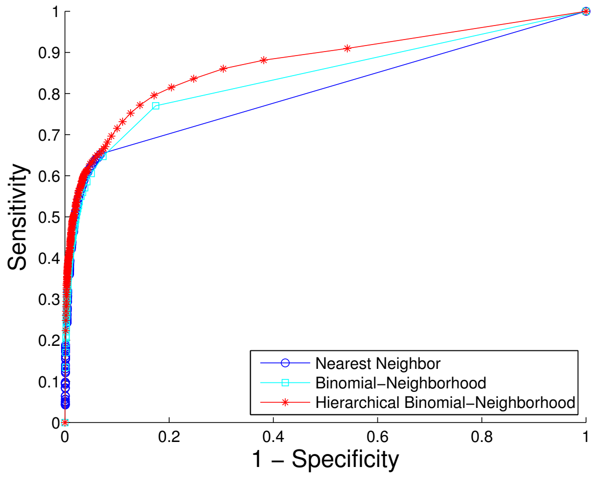

Figure 1: ROC curves by 5-fold cross-validation study.

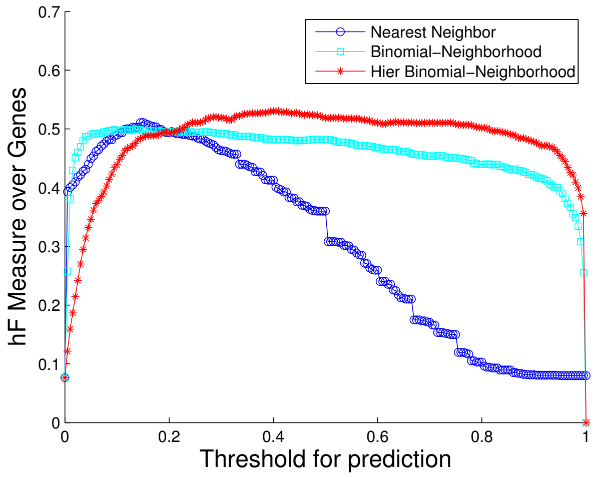

Figure 2: hF measure v.s. threshold.

8. **Hierarchy root: GO:0006950, response to stress**
Number of terms in hierarchy = 73
Number of terms predicted = 36
Number of genes predicted = 389

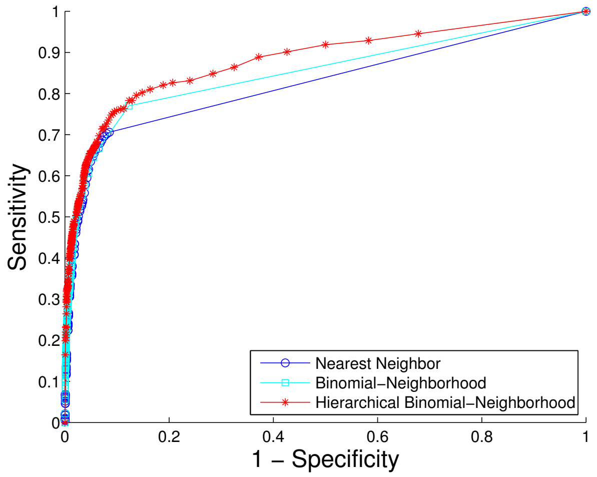

Figure 1: ROC curves by 5-fold cross-validation study.

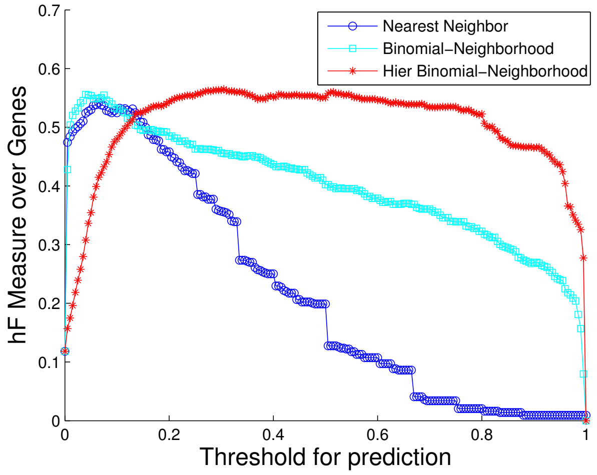

Figure 2: hF measure v.s. threshold.

9. **Hierarchy root: GO:0006996, organelle organization and biogenesis**
Number of terms in hierarchy = 122
Number of terms predicted = 68
Number of genes predicted = 992

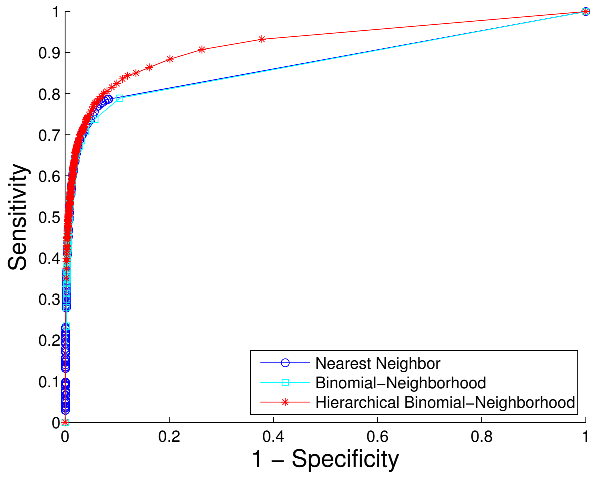

Figure 1: ROC curves by 5-fold cross-validation study.

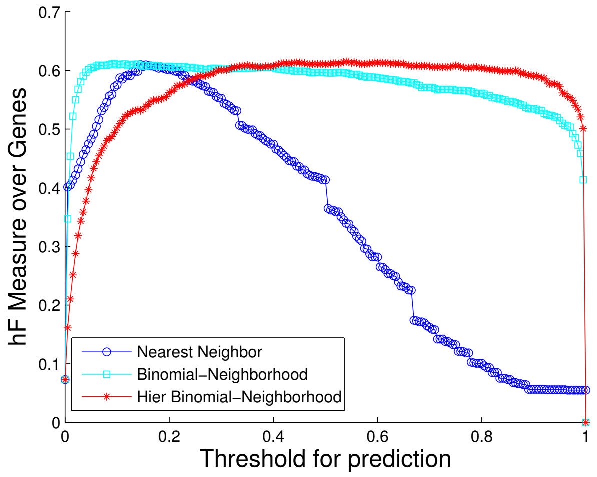

Figure 2: hF measure v.s. threshold.

10. **Hierarchy root: GO:0007001, chromosome organization and biogenesis (sensu Eukaryota)**
Number of terms in hierarchy = 32
Number of terms predicted = 23
Number of genes predicted = 356

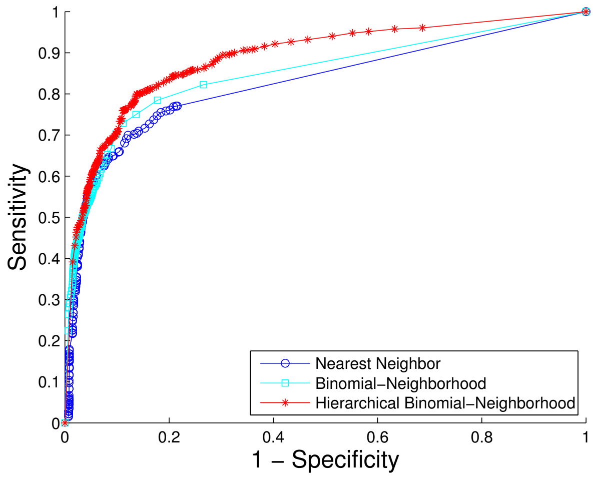

Figure 1: ROC curves by 5-fold cross-validation study.

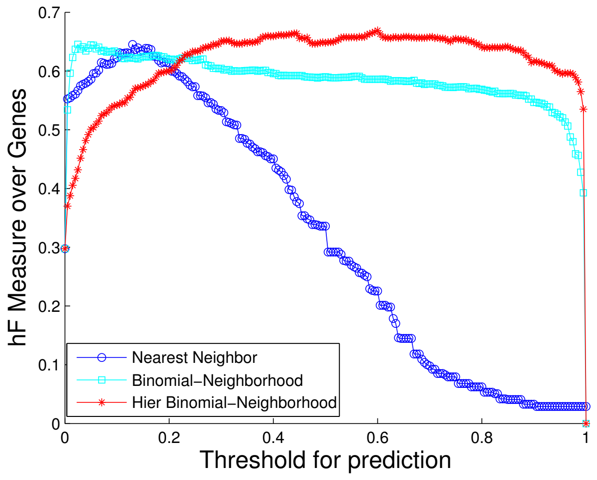

Figure 2: hF measure v.s. threshold.

11. **Hierarchy root: GO:0007049, cell cycle**
Number of terms in hierarchy = 97
Number of terms predicted = 63
Number of genes predicted = 399

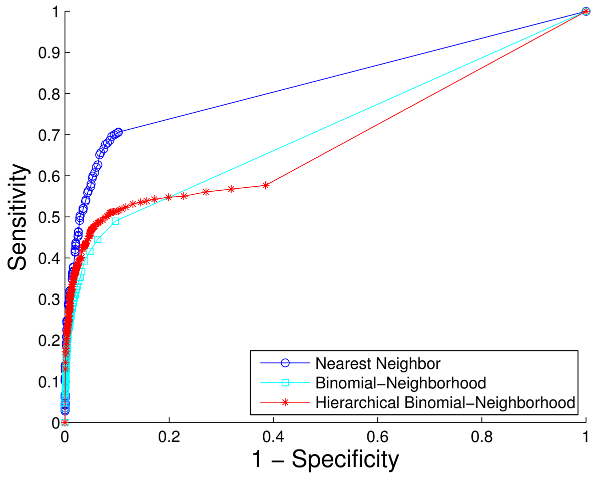

Figure 1: ROC curves by 5-fold cross-validation study.

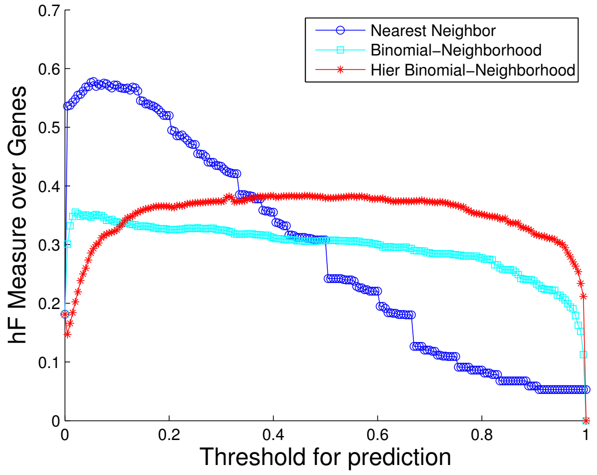

Figure 2: hF measure v.s. threshold.

12. **Hierarchy root: GO:0007275, multicellular organismal development**
Number of terms in hierarchy = 72
Number of terms predicted = 48
Number of genes predicted = 348

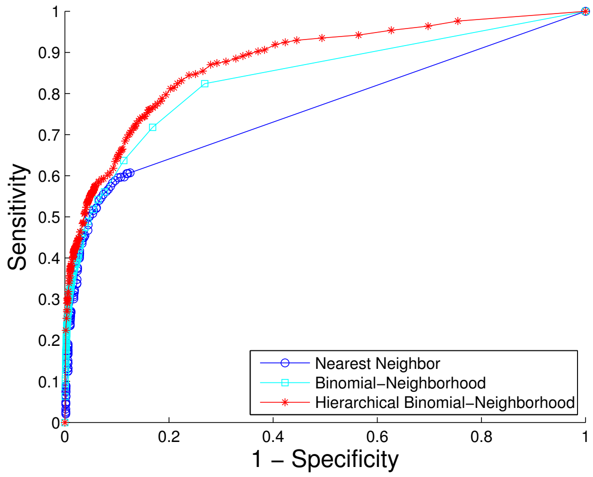

Figure 1: ROC curves by 5-fold cross-validation study.

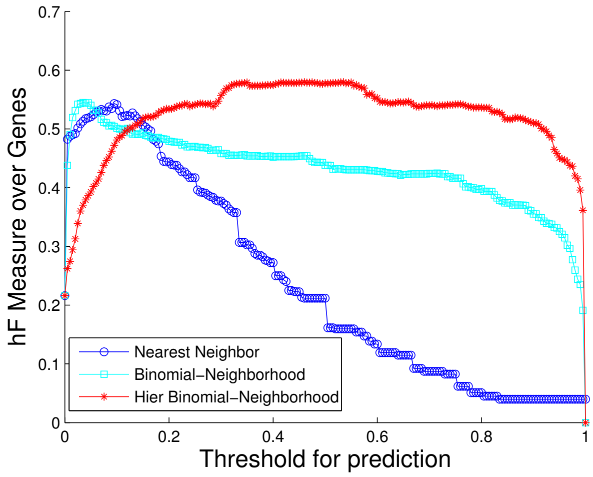

Figure 2: hF measure v.s. threshold.

13. **Hierarchy root: GO:0007582, physiological process**
Number of terms in hierarchy = 15
Number of terms predicted = 8
Number of genes predicted = 4052

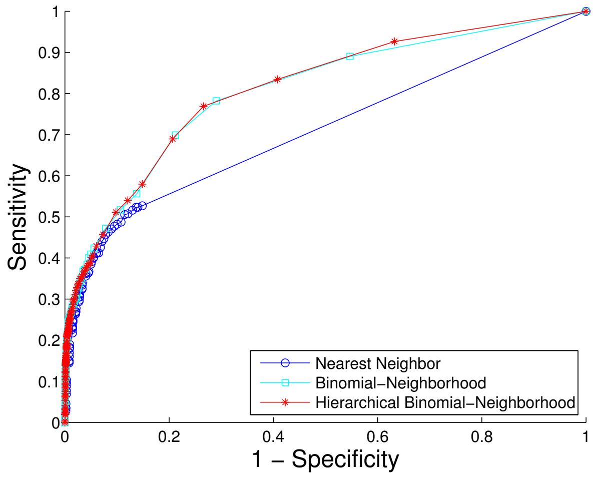

Figure 1: ROC curves by 5-fold cross-validation study.

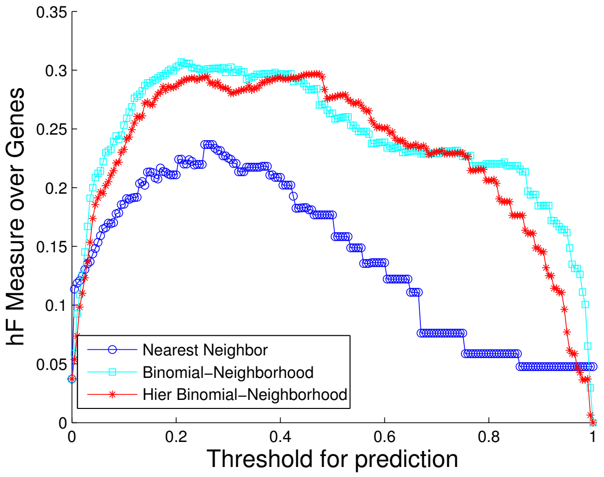

Figure 2: hF measure v.s. threshold.

14. **Hierarchy root: GO:0008150, biological process**
Number of terms in hierarchy = 70
Number of terms predicted = 41
Number of genes predicted = 5143

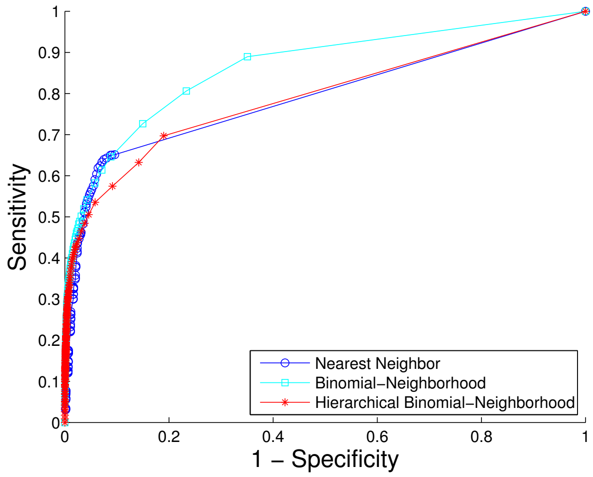

Figure 1: ROC curves by 5-fold cross-validation study.

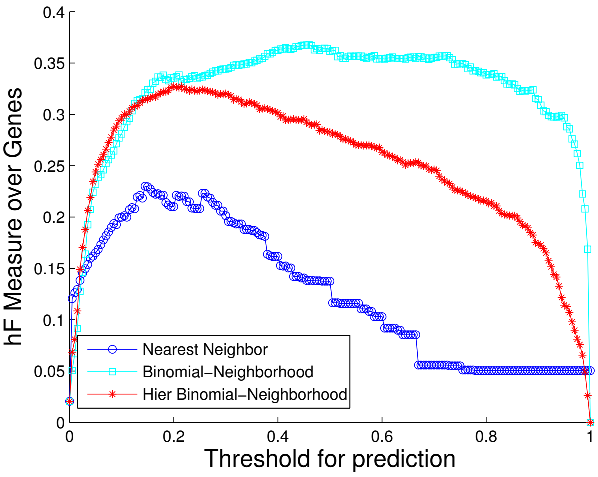

Figure 2: hF measure v.s. threshold.

15. **Hierarchy root: GO:0008152, metobalism**
Number of terms in hierarchy = 8
Number of terms predicted = 5
Number of genes predicted = 2842

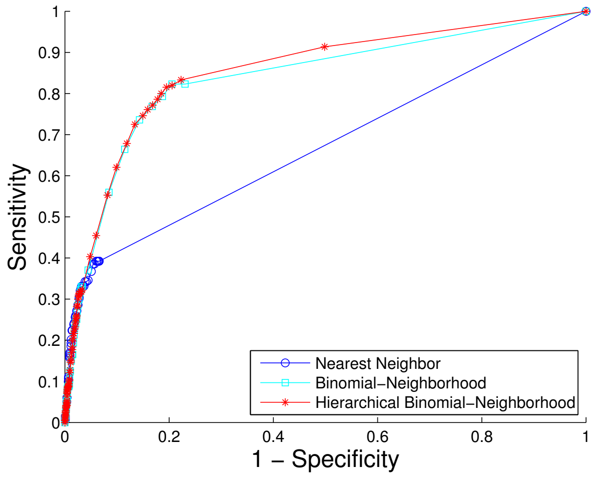

Figure 1: ROC curves by 5-fold cross-validation study.

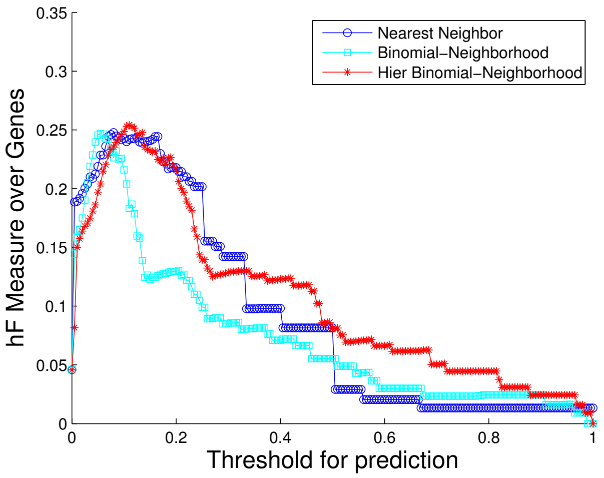

Figure 2: hF measure v.s. threshold.

16. **Hierarchy root: GO:0009056, catabolism**
Number of terms in hierarchy = 11
Number of terms predicted = 8
Number of genes predicted = 350

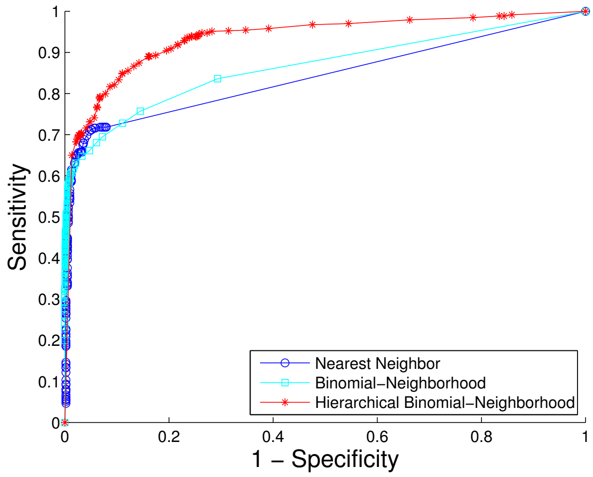

Figure 1: ROC curves by 5-fold cross-validation study.

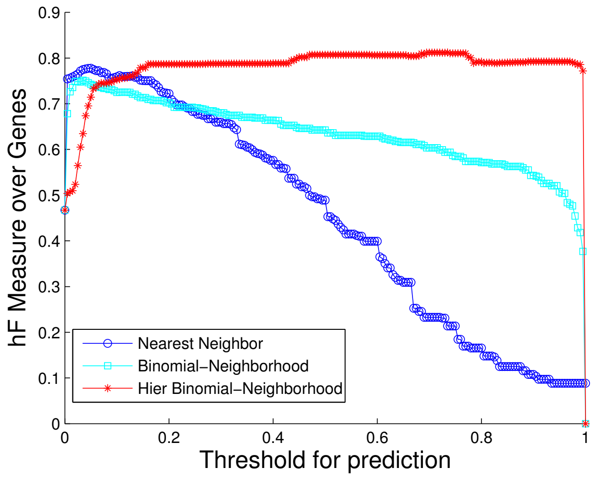

Figure 2: hF measure v.s. threshold.

17. **Hierarchy root: GO:0009058, biosynthesis**
Number of terms in hierarchy = 28
Number of terms predicted = 14
Number of genes predicted = 841

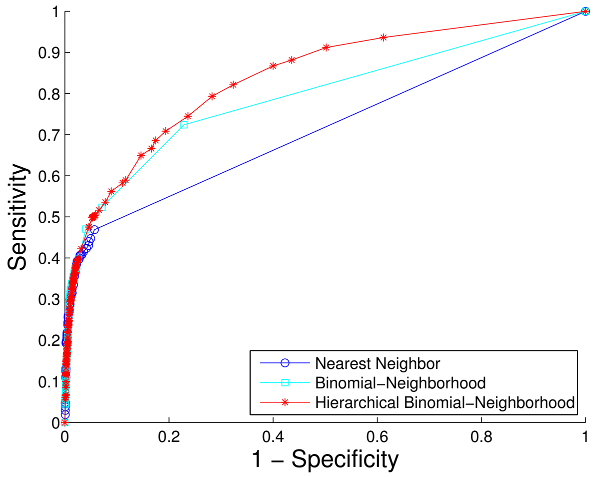

Figure 1: ROC curves by 5-fold cross-validation study.

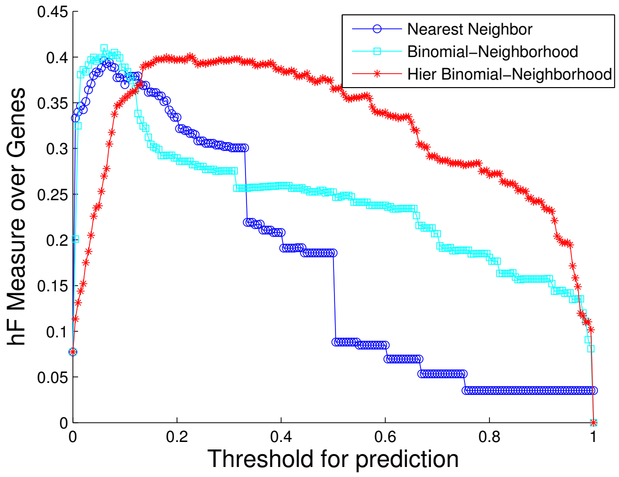

Figure 2: hF measure v.s. threshold.

18. **Hierarchy root: GO:0009059, macromolecule biosynthesis**
Number of terms in hierarchy = 28
Number of terms predicted = 20
Number of genes predicted = 523

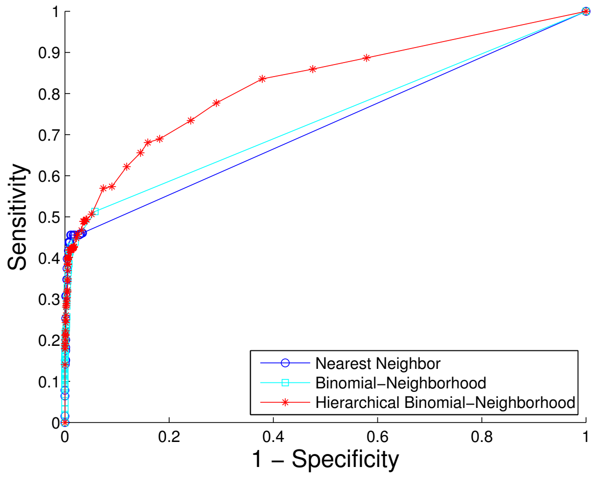

Figure 1: ROC curves by 5-fold cross-validation study.

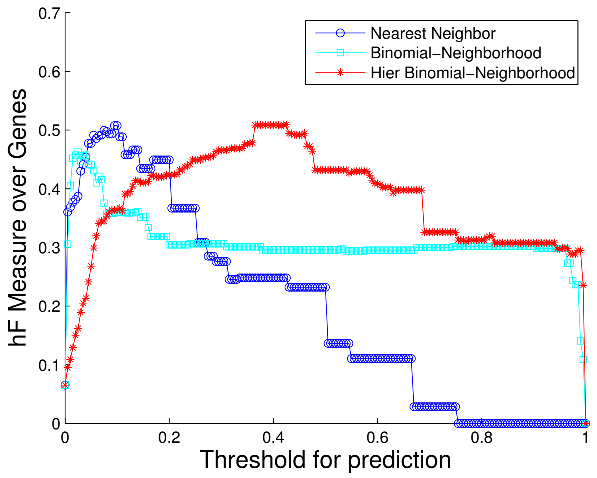

Figure 2: hF measure v.s. threshold.

19. **Hierarchy root: GO:0009987, cellular process**
Number of terms in hierarchy = 47
Number of terms predicted = 34
Number of genes predicted = 4029

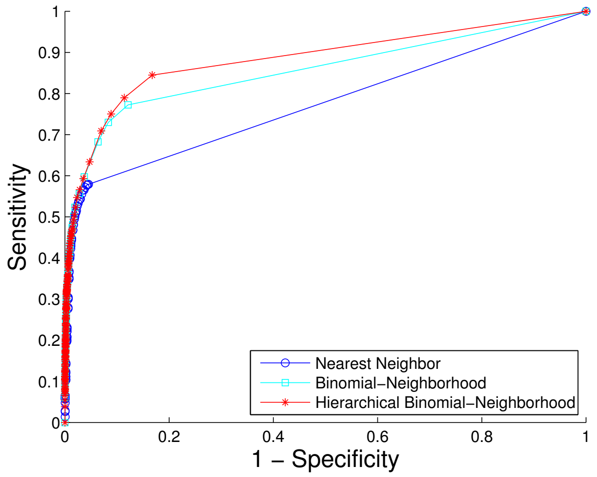

Figure 1: ROC curves by 5-fold cross-validation study.

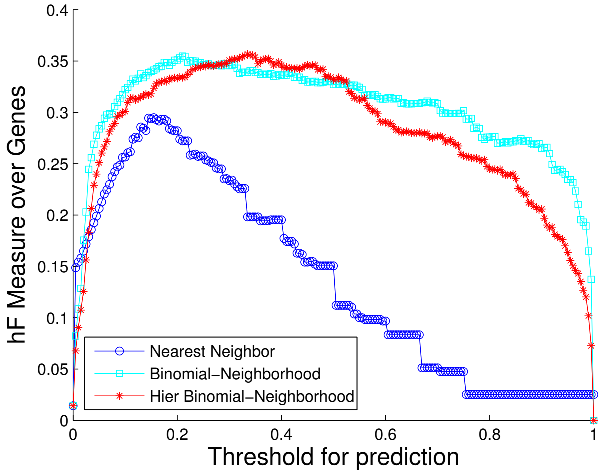

Figure 2: hF measure v.s. threshold.

20. **Hierarchy root: GO:0016043, cell organization and biogenesis**
Number of terms in hierarchy = 53
Number of terms predicted = 41
Number of genes predicted = 1554

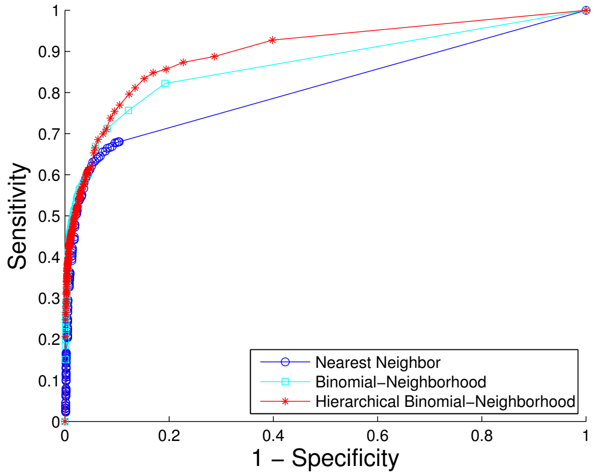

Figure 1: ROC curves by 5-fold cross-validation study.

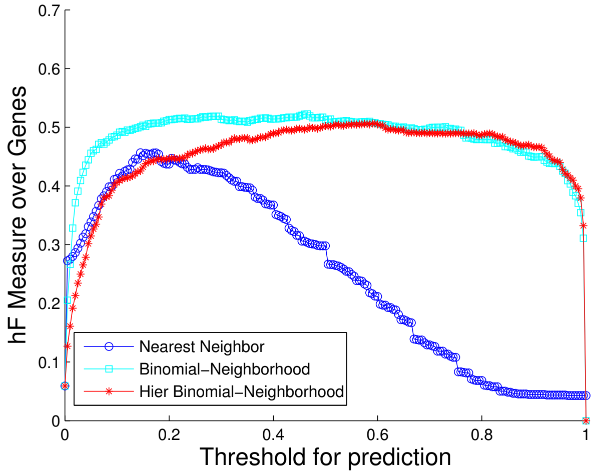

Figure 2: hF measure v.s. threshold.

21. **Hierarchy root: GO:0016070, RNA metabolism**
Number of terms in hierarchy = 57
Number of terms predicted = 23
Number of genes predicted = 441

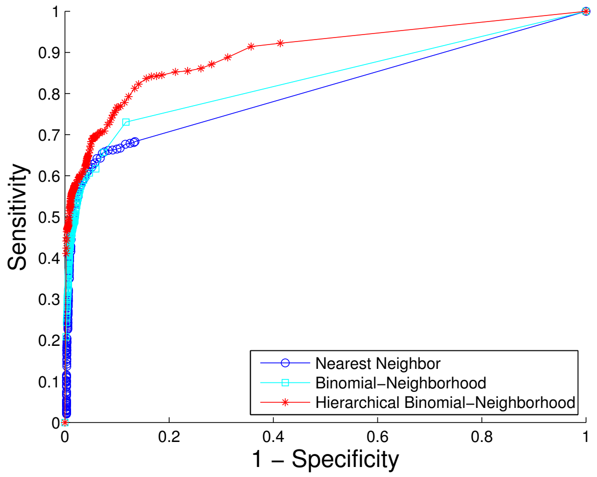

Figure 1: ROC curves by 5-fold cross-validation study.

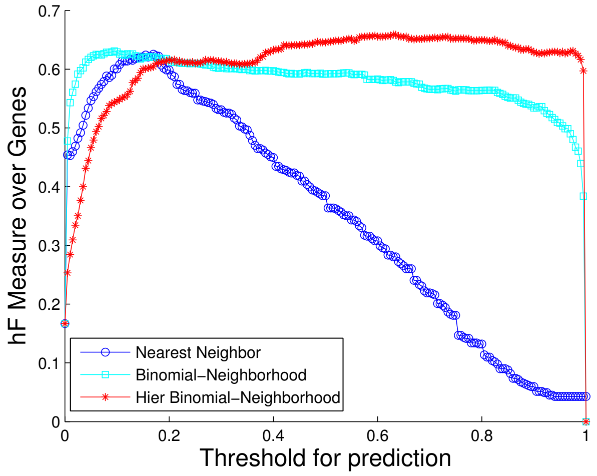

Figure 2: hF measure v.s. threshold.

22. **Hierarchy root: GO:0019219, regulation of nucleobase, nucleoside, nucleotide and nucleic acid metabolism**
Number of terms in hierarchy = 32
Number of terms predicted = 16
Number of genes predicted = 340

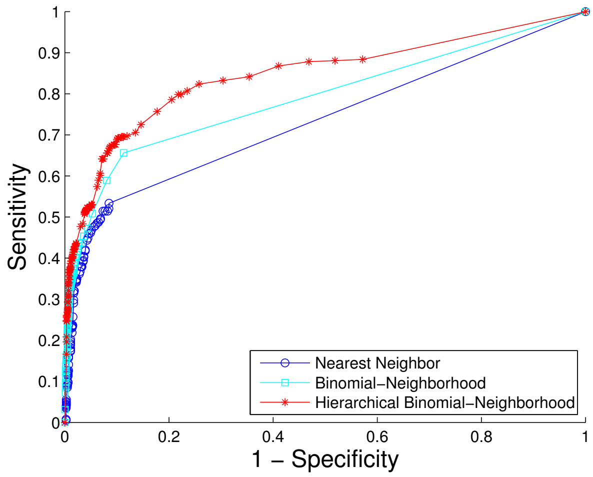

Figure 1: ROC curves by 5-fold cross-validation study.

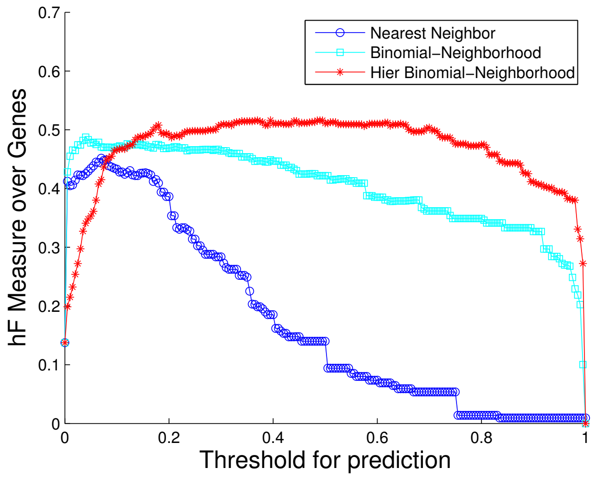

Figure 2: hF measure v.s. threshold.

23. **Hierarchy root: GO:0019222, regulation of metabolism**
Number of terms in hierarchy = 33
Number of terms predicted = 14
Number of genes predicted = 424

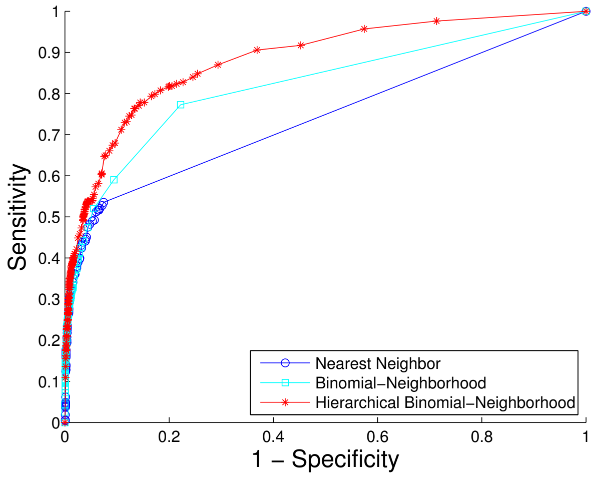

Figure 1: ROC curves by 5-fold cross-validation study.

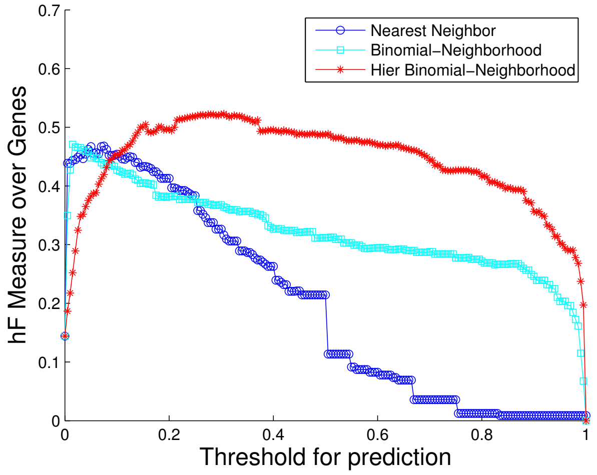

Figure 2: hF measure v.s. threshold.

24. **Hierarchy root: GO:0019538, protein metabolism**
Number of terms in hierarchy = 36
Number of terms predicted = 15
Number of genes predicted = 1105

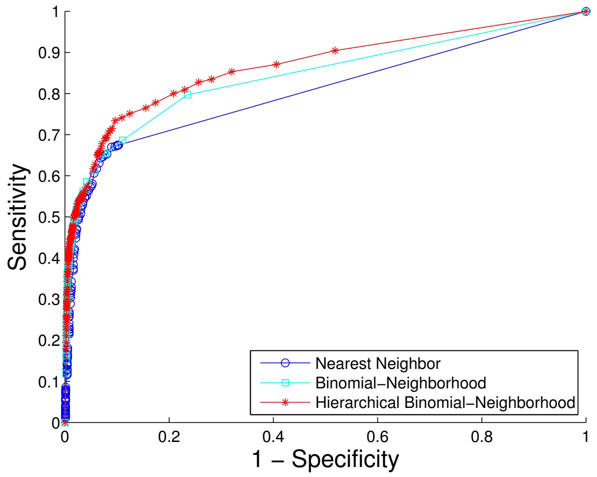

Figure 1: ROC curves by 5-fold cross-validation study.

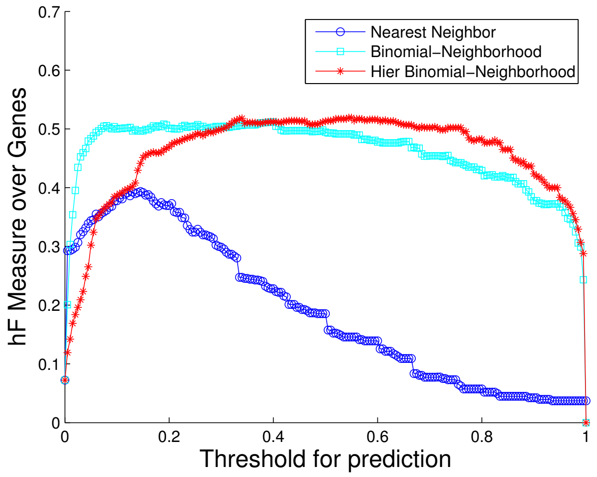

Figure 2: hF measure v.s. threshold.

25. **Hierarchy root: GO:0031323, regulation of cellular metabolism**
Number of terms in hierarchy = 33
Number of terms predicted = 17
Number of genes predicted = 399

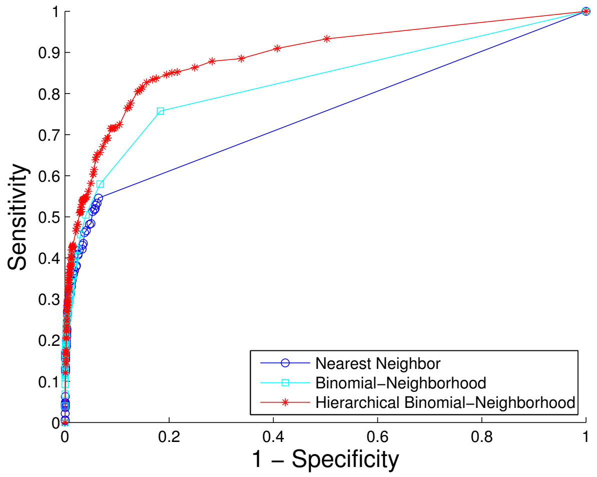

Figure 1: ROC curves by 5-fold cross-validation study.

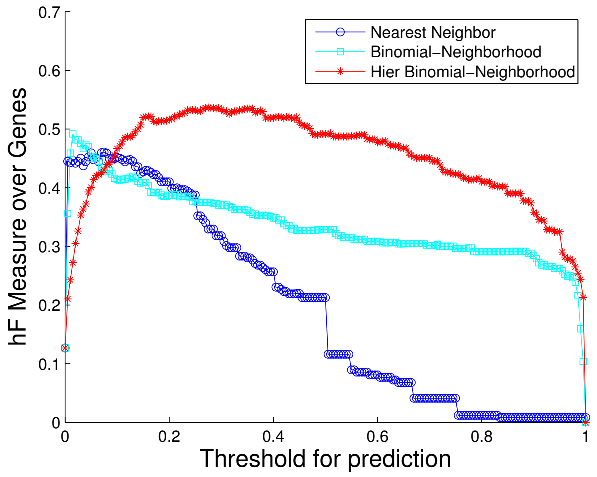

Figure 2: hF measure v.s. threshold.

26. **Hierarchy root: GO:0043170, macromolecule matabolism**
Number of terms in hierarchy = 73
Number of terms predicted = 36
Number of genes predicted = 2001

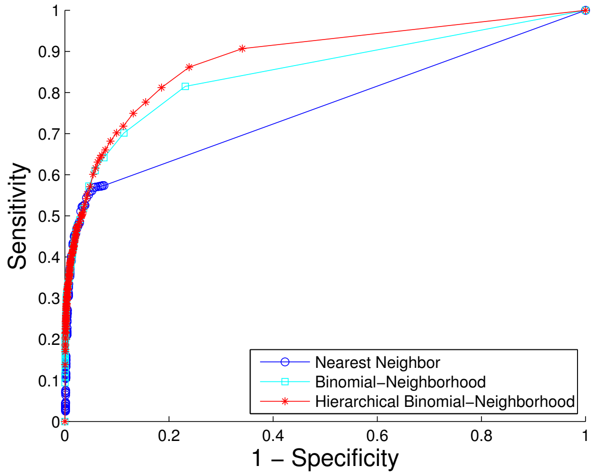

Figure 1: ROC curves by 5-fold cross-validation study.

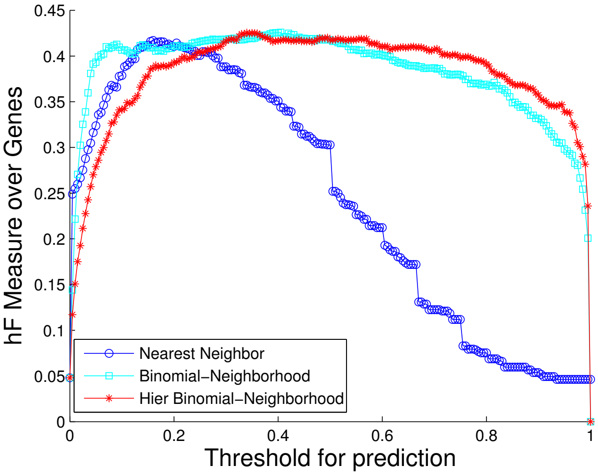

Figure 2: hF measure v.s. threshold.

27. **Hierarchy root: GO:0043283, biopolymer metabolism**
Number of terms in hierarchy = 53
Number of terms predicted = 36
Number of genes predicted = 1438

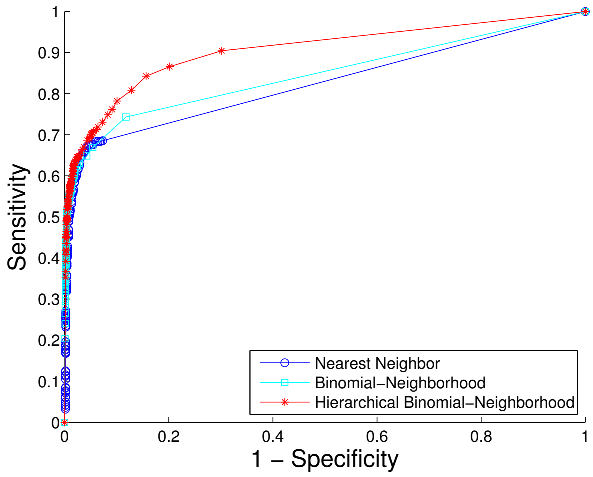

Figure 1: ROC curves by 5-fold cross-validation study.

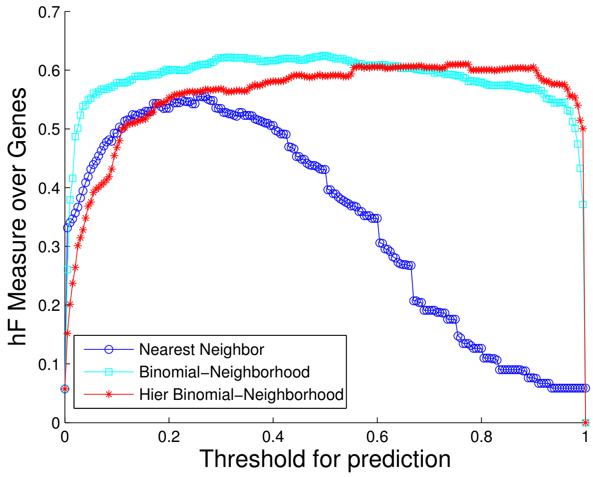

Figure 2: hF measure v.s. threshold.

28. **Hierarchy root: GO:0043412, biopolymer modification**
Number of terms in hierarchy = 22
Number of terms predicted = 12
Number of genes predicted = 491

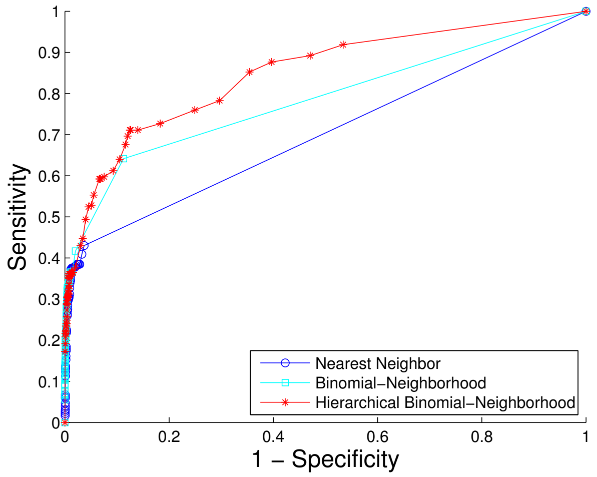

Figure 1: ROC curves by 5-fold cross-validation study.

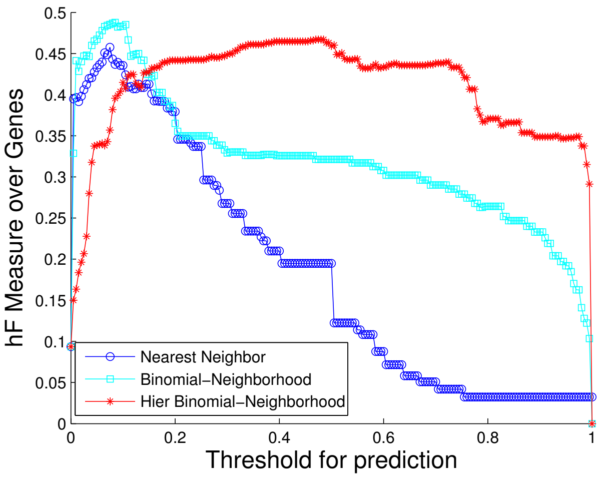

Figure 2: hF measure v.s. threshold.

29. **Hierarchy root: GO:0044237, cellular metabolism**
Number of terms in hierarchy = 266
Number of terms predicted = 149
Number of genes predicted = 2760

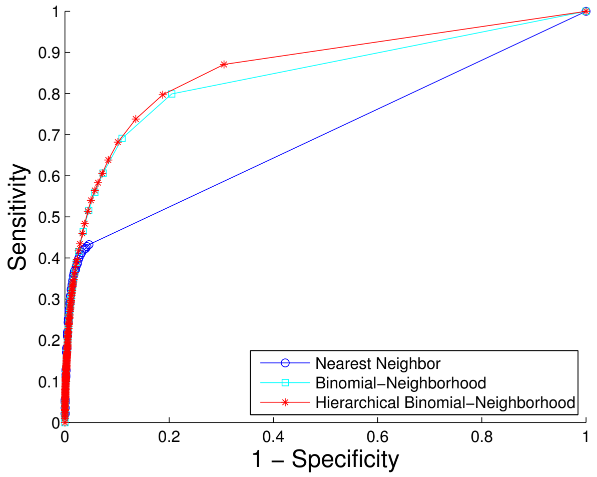

Figure 1: ROC curves by 5-fold cross-validation study.

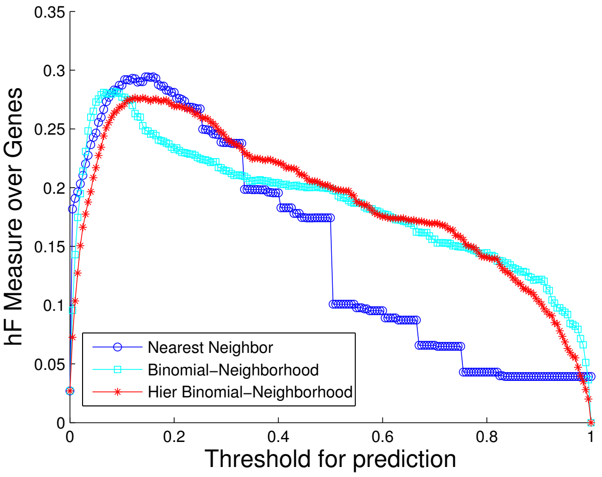

Figure 2: hF measure v.s. threshold.

30. **Hierarchy root: GO:0044238, primary metabolism**
Number of terms in hierarchy = 319
Number of terms predicted = 150
Number of genes predicted = 2600

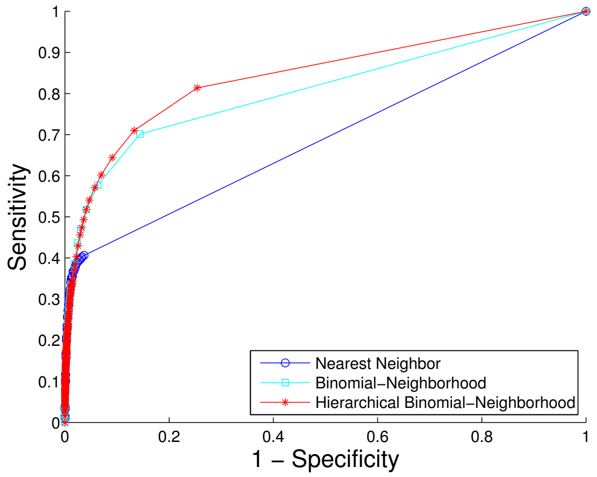

Figure 1: ROC curves by 5-fold cross-validation study.

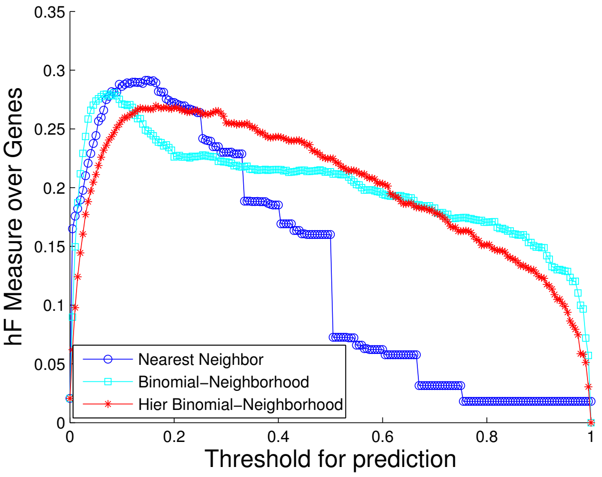

Figure 2: hF measure v.s. threshold.

31. **Hierarchy root: GO:0044248, cellular catabolism**
Number of terms in hierarchy = 154
Number of terms predicted = 51
Number of genes predicted = 338

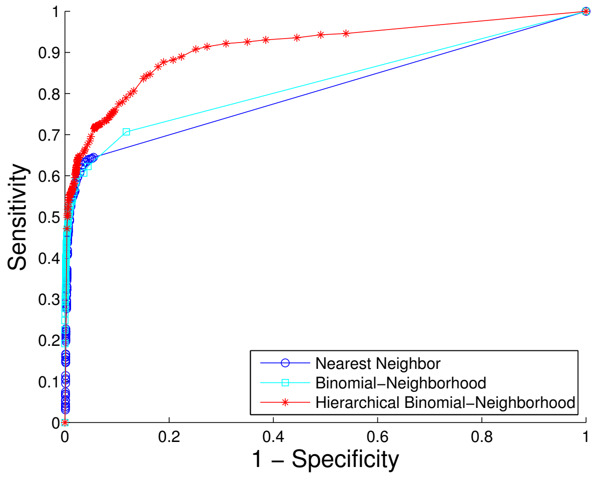

Figure 1: ROC curves by 5-fold cross-validation study.

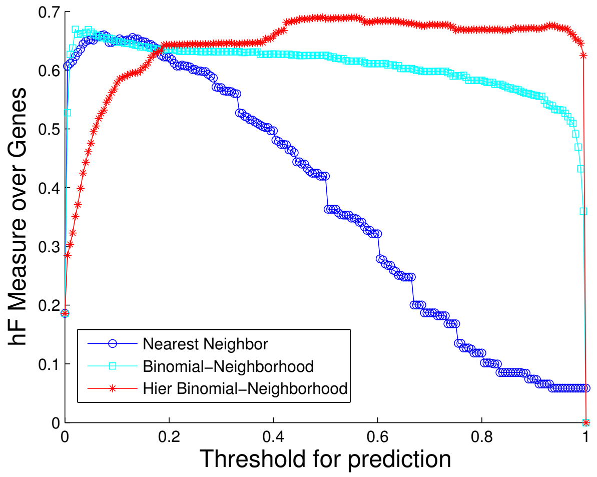

Figure 2: hF measure v.s. threshold.

32. **Hierarchy root: GO:0044249, cellular biosynthesis**
Number of terms in hierarchy = 148
Number of terms predicted = 67
Number of genes predicted = 768

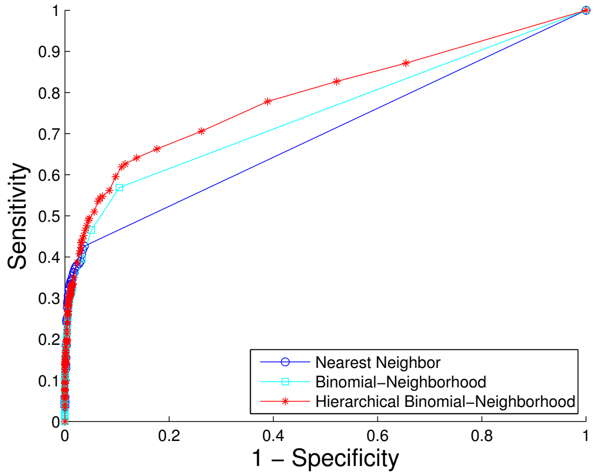

Figure 1: ROC curves by 5-fold cross-validation study.

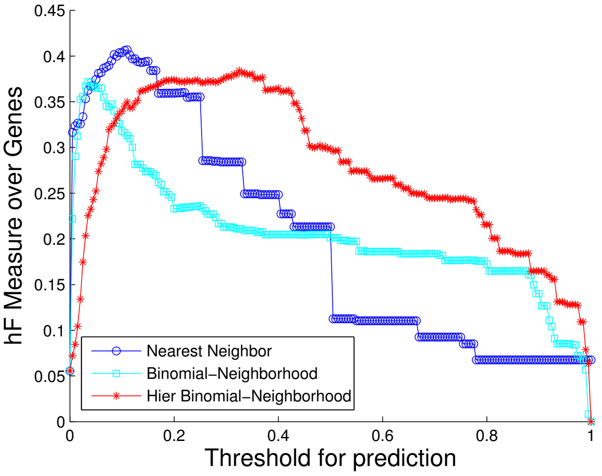

Figure 2: hF measure v.s. threshold.

33. **Hierarchy root: GO:0044260, cellular macromolecule metabolism**
Number of terms in hierarchy = 32
Number of terms predicted = 20
Number of genes predicted = 1031

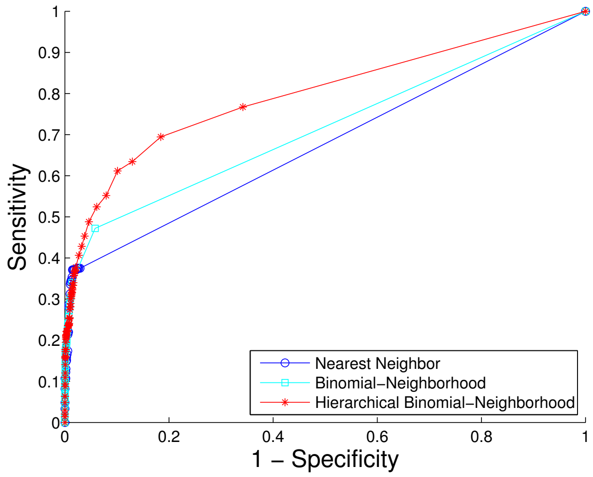

Figure 1: ROC curves by 5-fold cross-validation study.

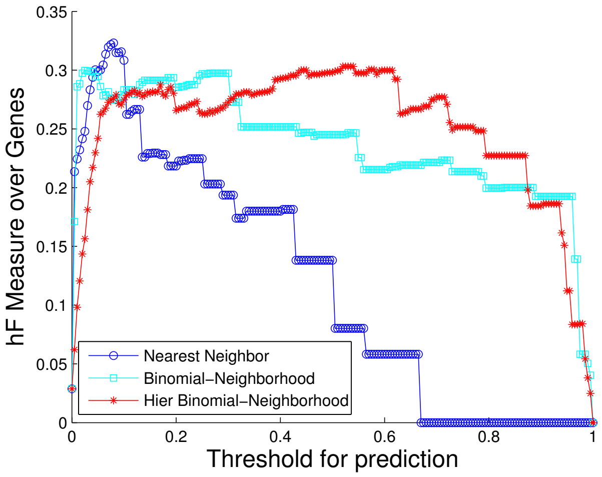

Figure 2: hF measure v.s. threshold.

34. **Hierarchy root: GO:0044267, cellular protein metabolism**
Number of terms in hierarchy = 49
Number of terms predicted = 22
Number of genes predicted = 986

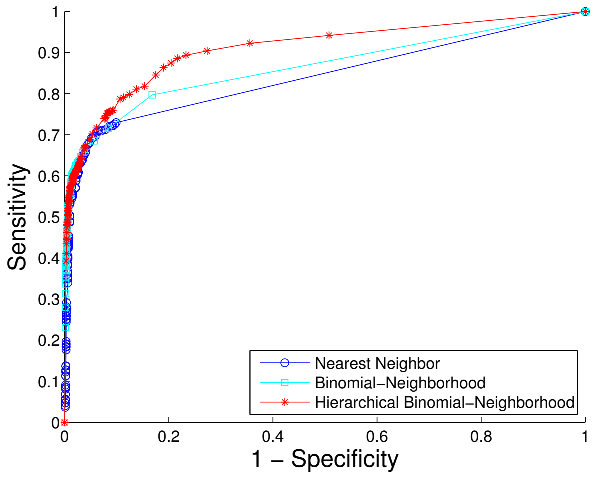

Figure 1: ROC curves by 5-fold cross-validation study.

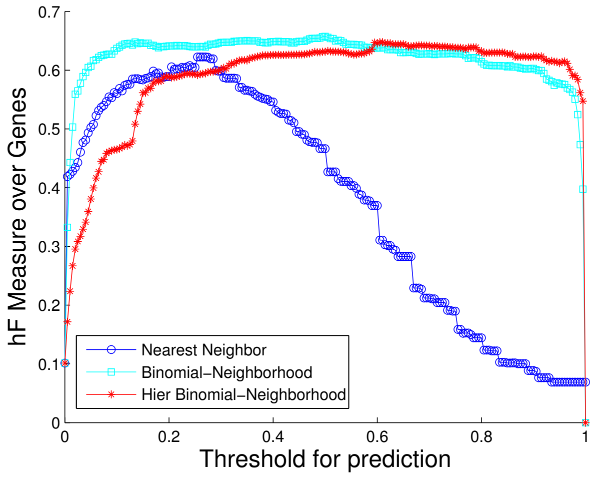

Figure 2: hF measure v.s. threshold.

35. **Hierarchy root: GO:0045449, regulation of transcription**
Number of terms in hierarchy = 43
Number of terms predicted = 27
Number of genes predicted = 309

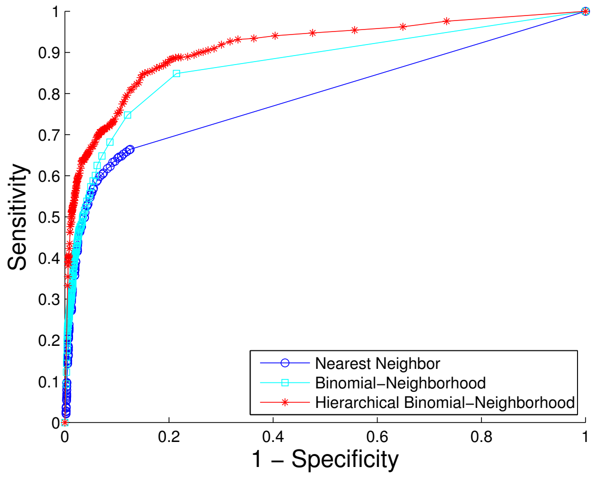

Figure 1: ROC curves by 5-fold cross-validation study.

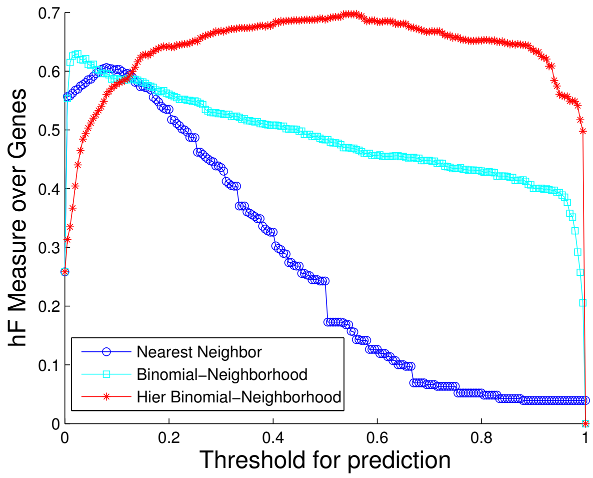

Figure 2: hF measure v.s. threshold.

36. **Hierarchy root: GO:0046907, intracellular transport**
Number of terms in hierarchy = 92
Number of terms predicted = 54
Number of genes predicted = 491

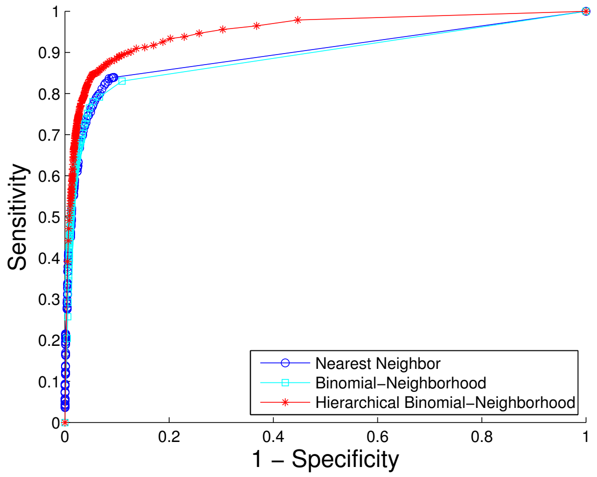

Figure 1: ROC curves by 5-fold cross-validation study.

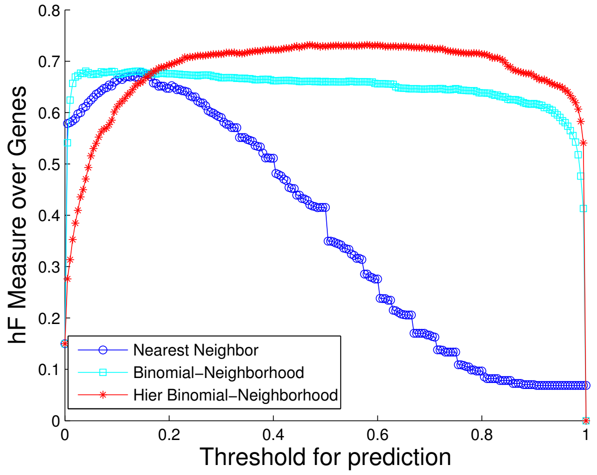

Figure 2: hF measure v.s. threshold.

37. **Hierarchy root: GO:0050789, regulation of biological process**
Number of terms in hierarchy = 42
Number of terms predicted = 19
Number of genes predicted = 618

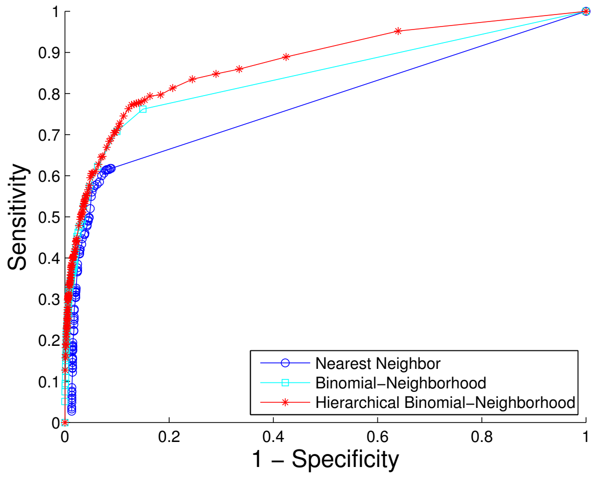

Figure 1: ROC curves by 5-fold cross-validation study.

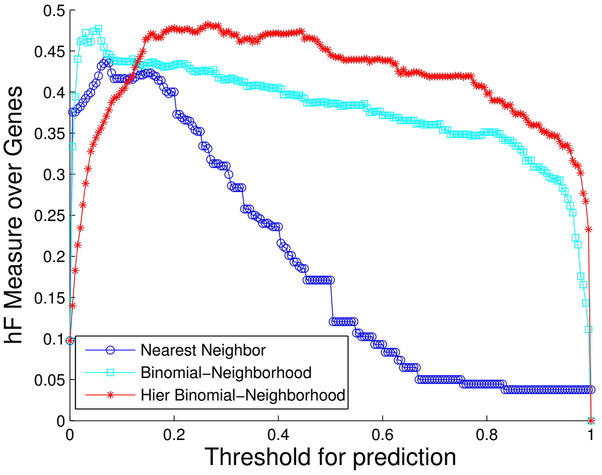

Figure 2: hF measure v.s. threshold.

38. **Hierarchy root: GO:0050791, regulation of physiological process**
Number of terms in hierarchy = 3
Number of terms predicted = 3
Number of genes predicted = 608

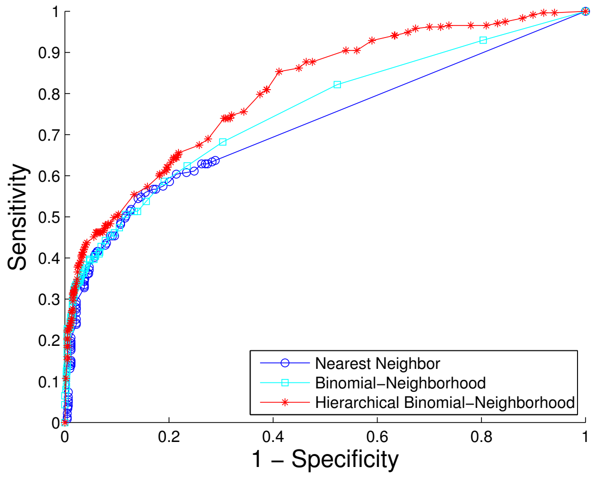

Figure 1: ROC curves by 5-fold cross-validation study.

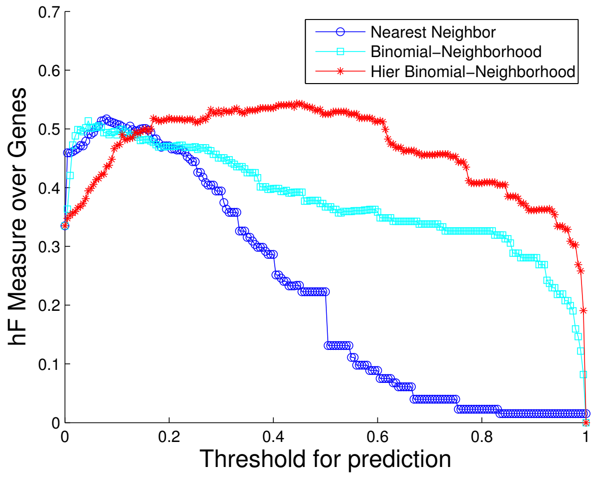

Figure 2: hF measure v.s. threshold.

39. **Hierarchy root: GO:0050794, regulation of cellular process**
Number of terms in hierarchy = 7
Number of terms predicted = 3
Number of genes predicted = 592

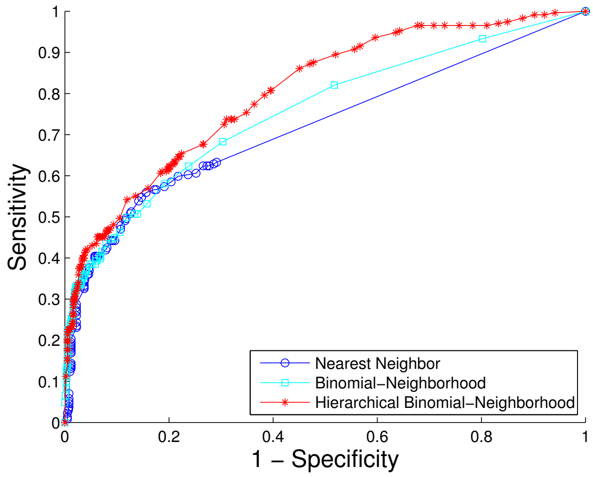

Figure 1: ROC curves by 5-fold cross-validation study.

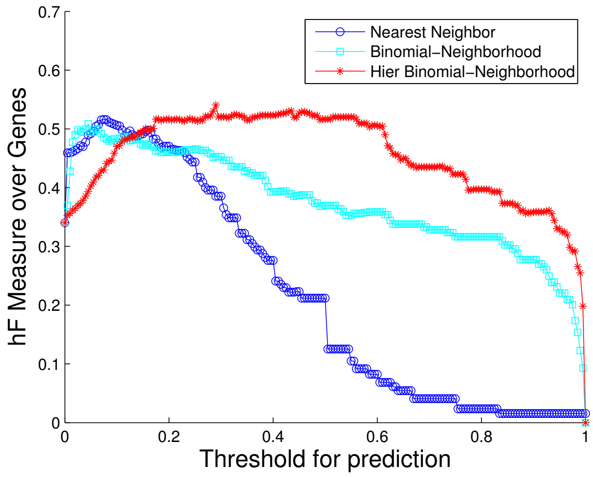

Figure 2: hF measure v.s. threshold.

40. **Hierarchy root: GO:0050875, cellular physiological process**
Number of terms in hierarchy = 83
Number of terms predicted = 59
Number of genes predicted = 4000

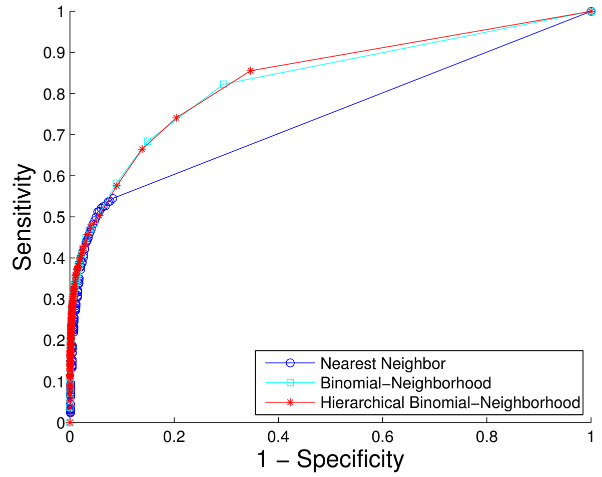

Figure 1: ROC curves by 5-fold cross-validation study.

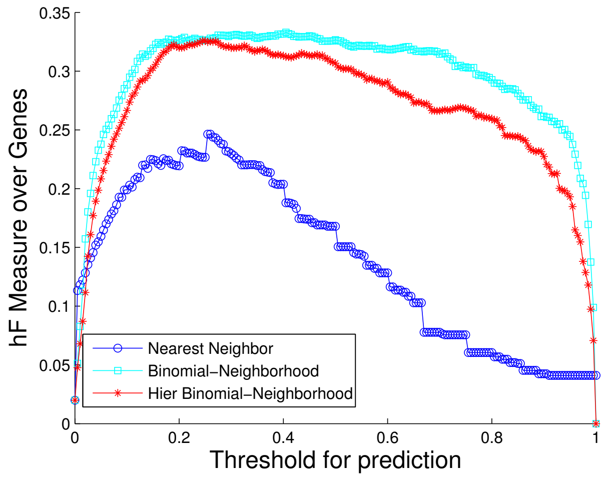

Figure 2: hF measure v.s. threshold.

41. **Hierarchy root: GO:0050896, response to stimulus**
Number of terms in hierarchy = 72
Number of terms predicted = 40
Number of genes predicted = 536

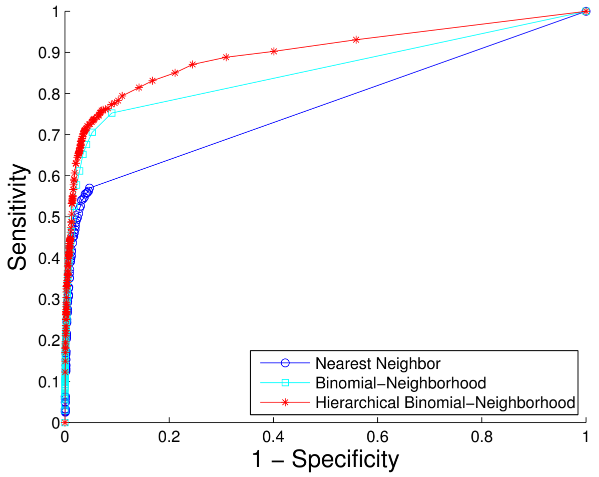

Figure 1: ROC curves by 5-fold cross-validation study.

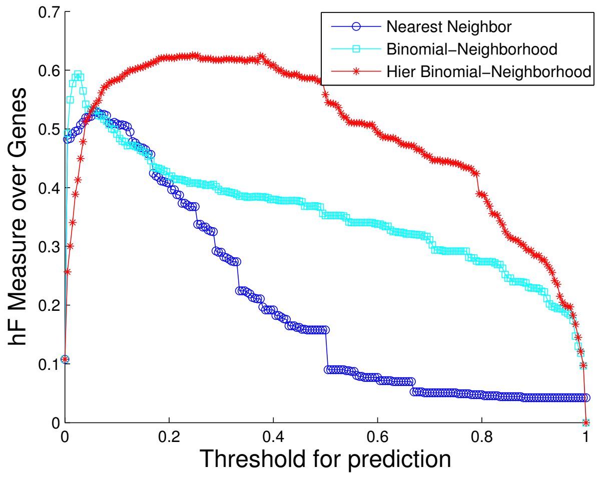

Figure 2: hF measure v.s. threshold.

42. **Hierarchy root: GO:0051179, localization**
Number of terms in hierarchy = 14
Number of terms predicted = 9
Number of genes predicted = 964

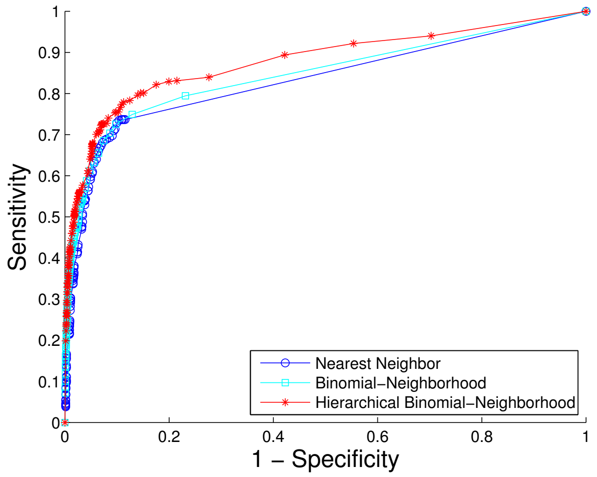

Figure 1: ROC curves by 5-fold cross-validation study.

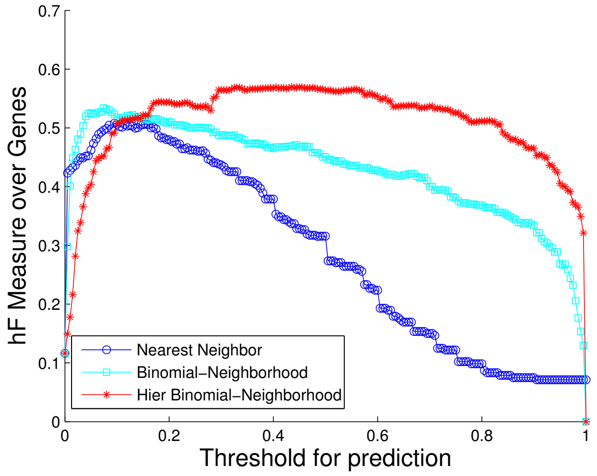

Figure 2: hF measure v.s. threshold.

43. **Hierarchy root: GO:0051234, establishment of localization**
Number of terms in hierarchy = 8
Number of terms predicted = 6
Number of genes predicted = 923

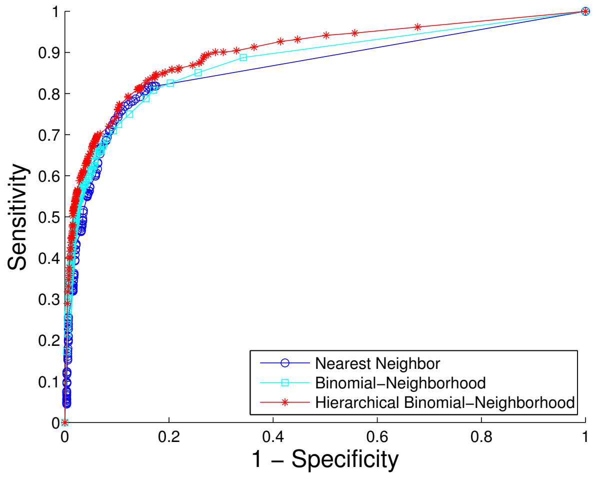

Figure 1: ROC curves by 5-fold cross-validation study.

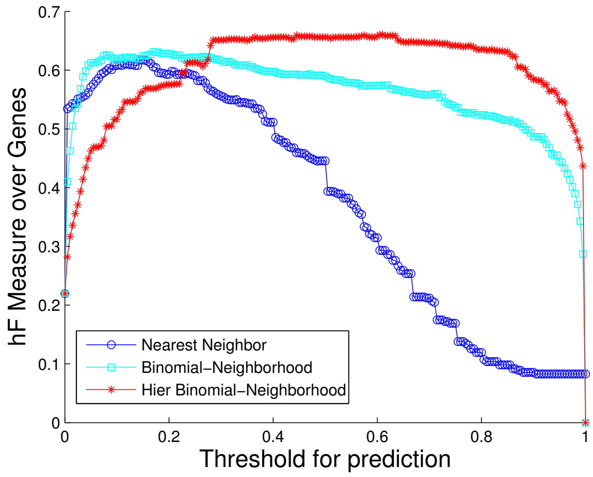

Figure 2: hF measure v.s. threshold.

44. **Hierarchy root: GO:0051244, regulation of cellular physiological process**
Number of terms in hierarchy = 95
Number of terms predicted = 34
Number of genes predicted = 591

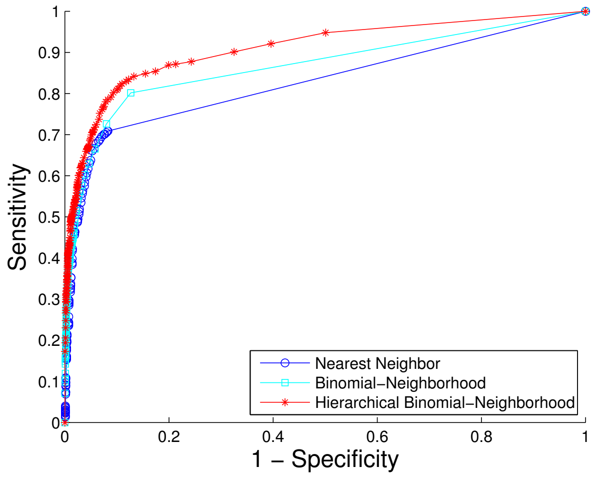

Figure 1: ROC curves by 5-fold cross-validation study.

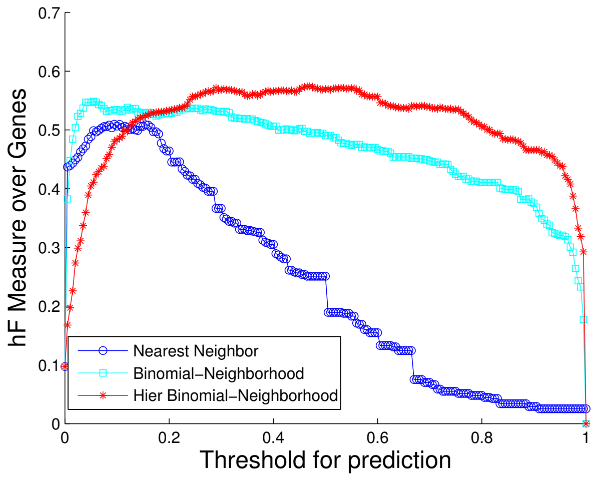

Figure 2: hF measure v.s. threshold.

45. **Hierarchy root: GO:0051276, chromosome organization and biogenesis**
Number of terms in hierarchy = 6
Number of terms predicted = 3
Number of genes predicted = 367

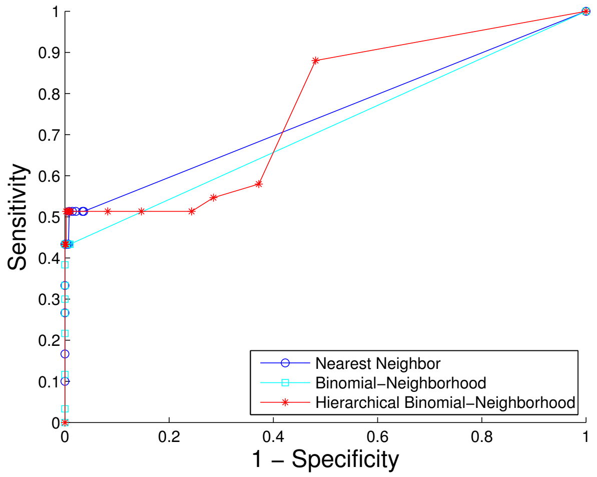

Figure 1: ROC curves by 5-fold cross-validation study.

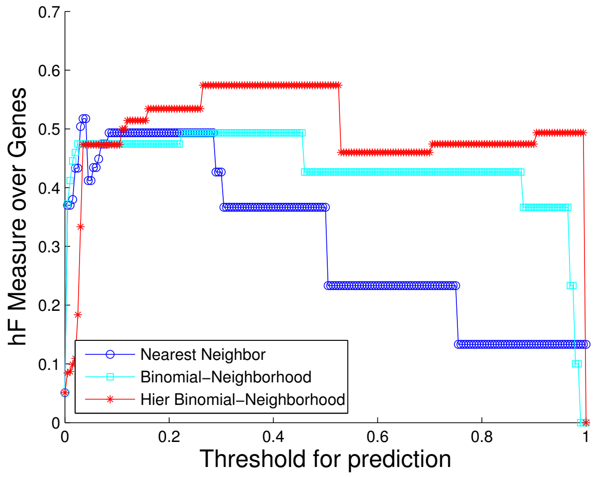

Figure 2: hF measure v.s. threshold.

46. **Hierarchy root: GO:0051641, cellular localization**
Number of terms in hierarchy = 12
Number of terms predicted = 6
Number of genes predicted = 527

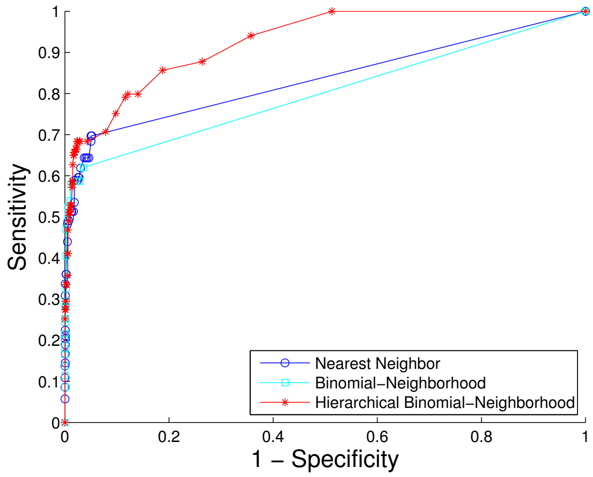

Figure 1: ROC curves by 5-fold cross-validation study.

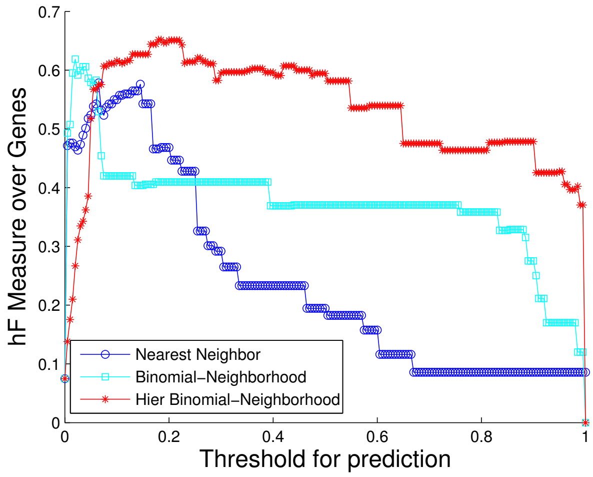

Figure 2: hF measure v.s. threshold.

47. **Hierarchy root: GO:0051649, establishment of cellular localization**
Number of terms in hierarchy = 7
Number of terms predicted = 7
Number of genes predicted = 500

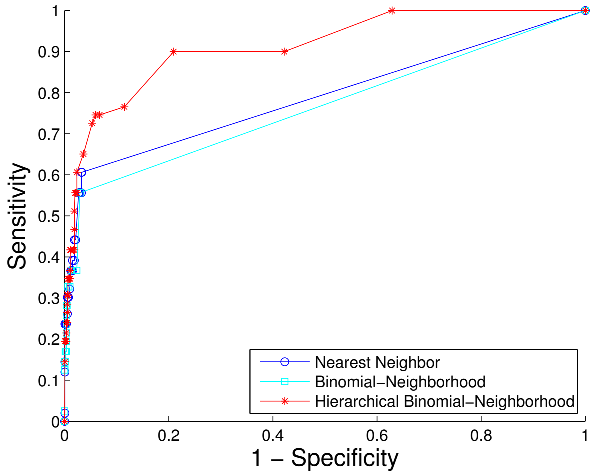

Figure 1: ROC curves by 5-fold cross-validation study.

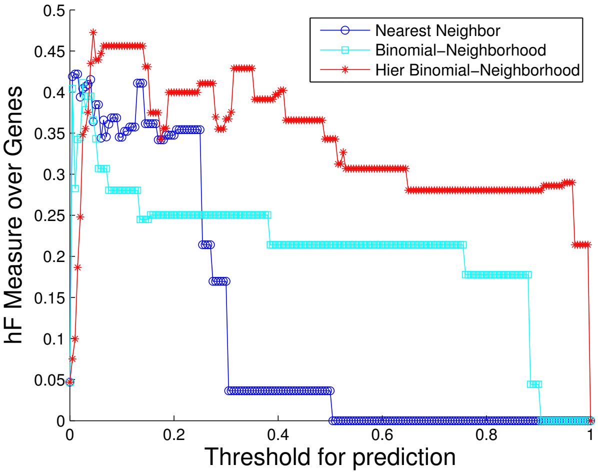

Figure 2: hF measure v.s. threshold.
