# Supplementary material for: Integration of relational and hierarchical network information for protein function prediction
Source: BMC Bioinformatics. 2008 Aug 22;9:350. doi: 10.1186/1471-2105-9-350 (PMC2535605; doi:10.1186/1471-2105-9-350)

Integration of Relational and Hierarchical Network Information for Protein Function Prediction

Additional file - 2

Xiaoyu Jiang, Naoki Nariai, Martin Steffen, Simon Kasif, Eric D. Kolaczyk

---

Colors: HBN (red); BN (light blue); NN (blue).

1. **Hierarchy root: GO:0006139, nucleobase, nucleoside, nucleotide and nucleic acid metabolism**
   Number of terms in hierarchy = 154
   Number of terms predicted = 58
   Number of genes predicted = 31

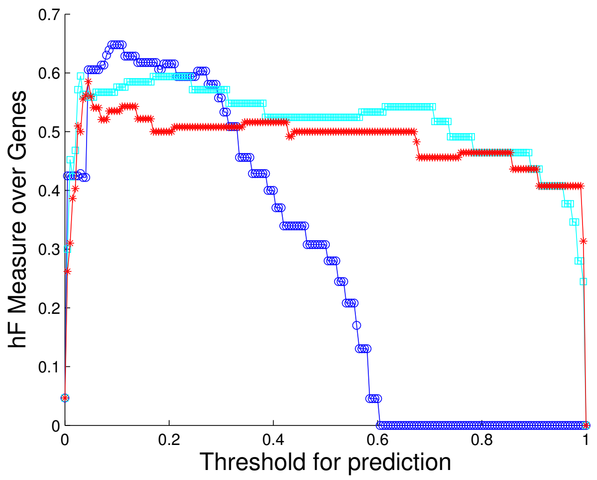

2. **Hierarchy root: GO:0008150, biological process**
   Number of terms in hierarchy = 70
   Number of terms predicted = 40
   Number of genes predicted = 58

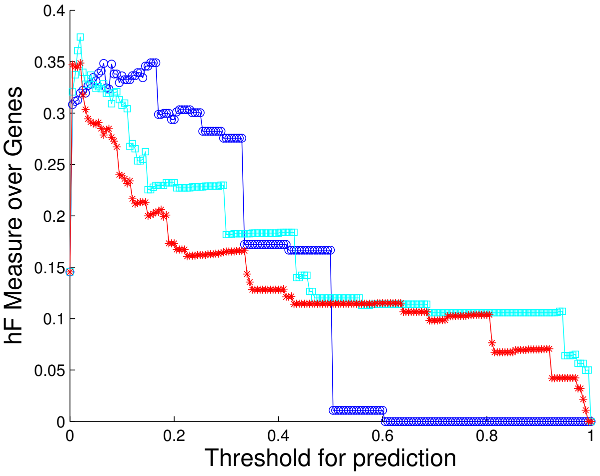

3. **Hierarchy root: GO:0009987, cellular process**
   Number of terms in hierarchy = 47
   Number of terms predicted = 33
   Number of genes predicted = 74

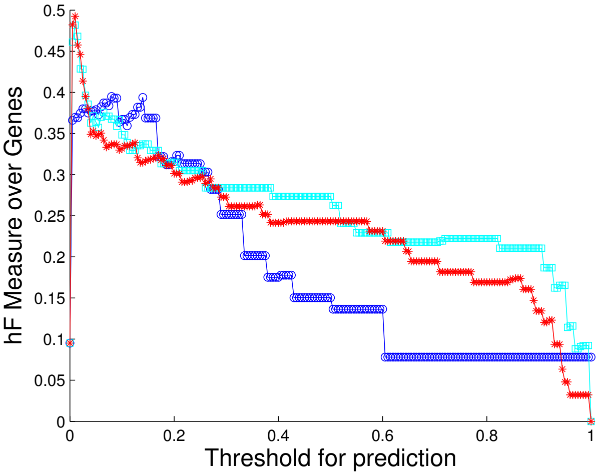

4. **Hierarchy root: GO:0016043, cell organization and biogenesis**
   Number of terms in hierarchy = 53
   Number of terms predicted = 40
   Number of genes predicted = 34

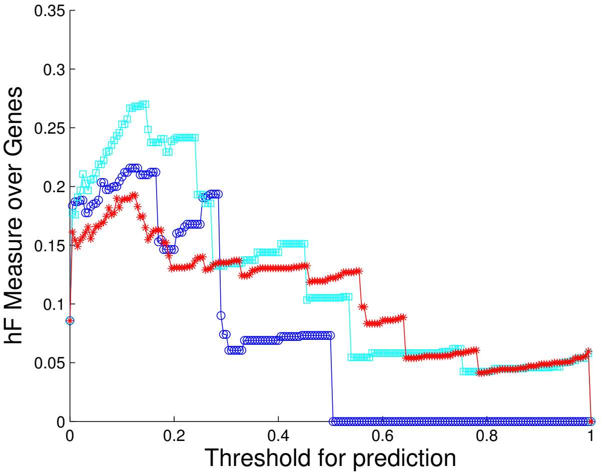

5. **Hierarchy root: GO:0019219, regulation of nucleobase, nucleoside, nucleotide and nucleic acid metabolism**
   Number of terms in hierarchy = 32
   Number of terms predicted = 15
   Number of genes predicted = 8

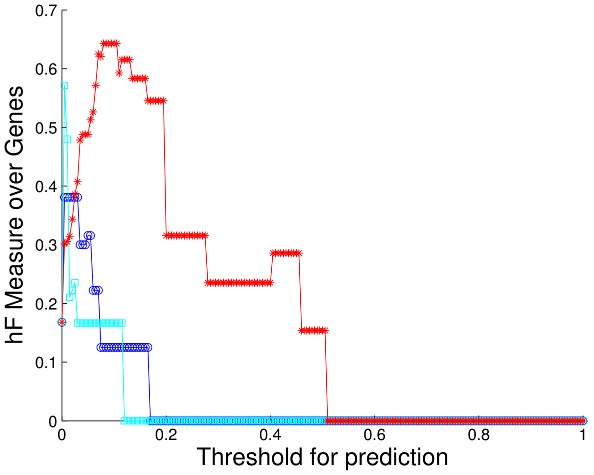

6. **Hierarchy root: GO:0019222, regulation of metabolism**
   Number of terms in hierarchy = 33
   Number of terms predicted = 13
   Number of genes predicted = 11

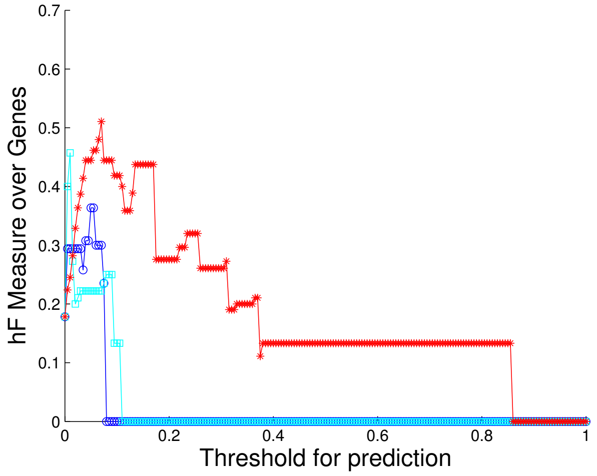

7. **Hierarchy root: GO:0019538, protein metabolism**
   Number of terms in hierarchy = 36
   Number of terms predicted = 14
   Number of genes predicted = 14

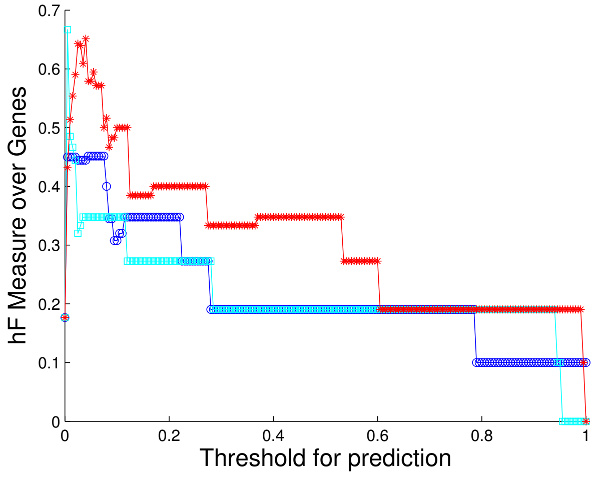

8. **Hierarchy root: GO:0031323, regulation of cellular metabolism**
   Number of terms in hierarchy = 33
   Number of terms predicted = 16
   Number of genes predicted = 10

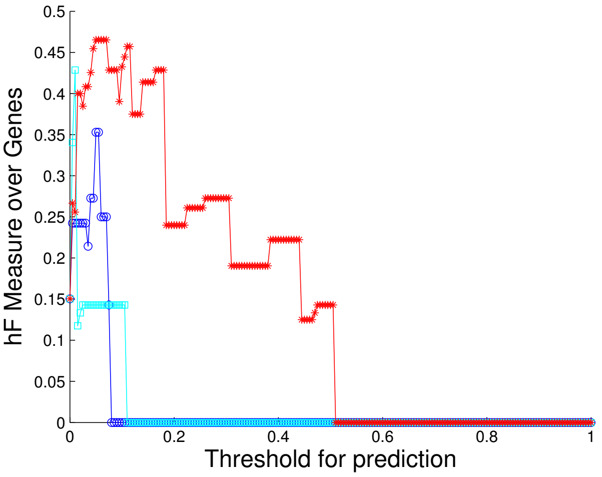

9. **Hierarchy root: GO:0043170, macromolecule matabolism**
   Number of terms in hierarchy = 73
   Number of terms predicted = 35
   Number of genes predicted = 20

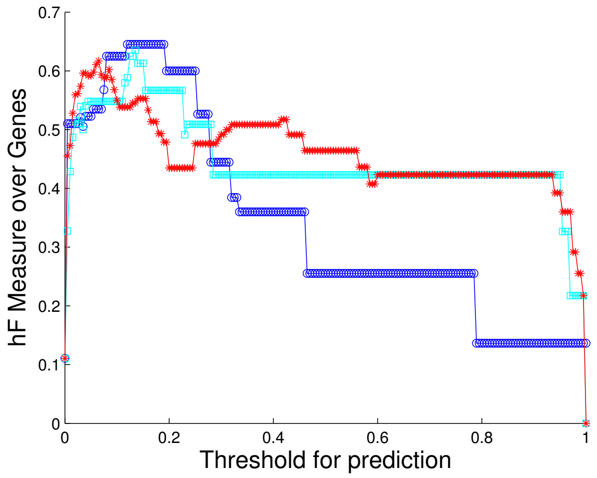

10. **Hierarchy root: GO:0043283, biopolymer metabolism**
Number of terms in hierarchy = 53
Number of terms predicted = 35
Number of genes predicted = 12

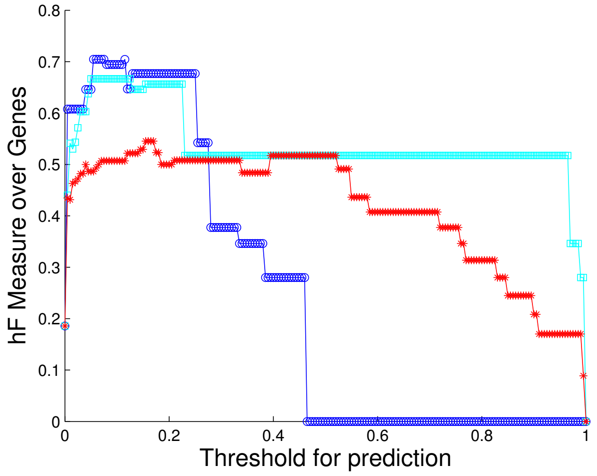

11. **Hierarchy root: GO:0044237, cellular metabolism**
Number of terms in hierarchy = 266
Number of terms predicted = 148
Number of genes predicted = 51

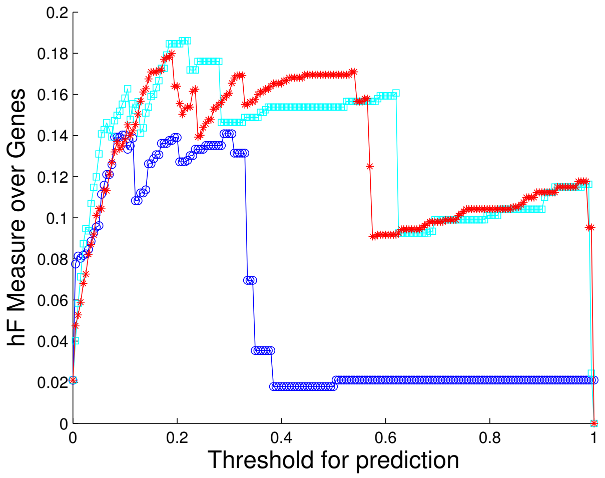

12. **Hierarchy root: GO:0044238, primary metabolism**
Number of terms in hierarchy = 266
Number of terms predicted = 149
Number of genes predicted = 8

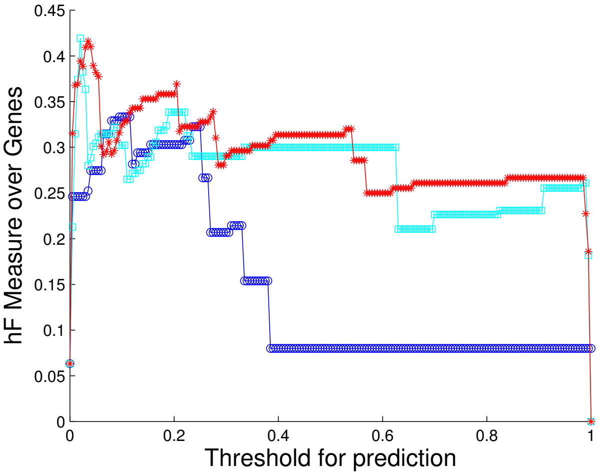

13. **Hierarchy root: GO:0050789, regulation of biological process**
Number of terms in hierarchy = 42
Number of terms predicted = 18
Number of genes predicted = 27

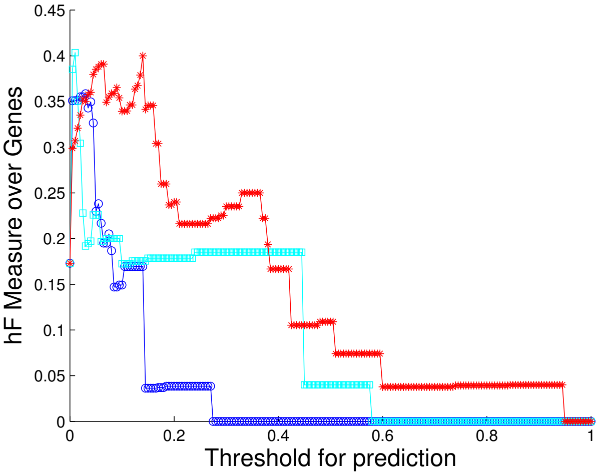

14. **Hierarchy root: GO:0050794, regulation of cellular process**
Number of terms in hierarchy = 7
Number of terms predicted = 2
Number of genes predicted = 21

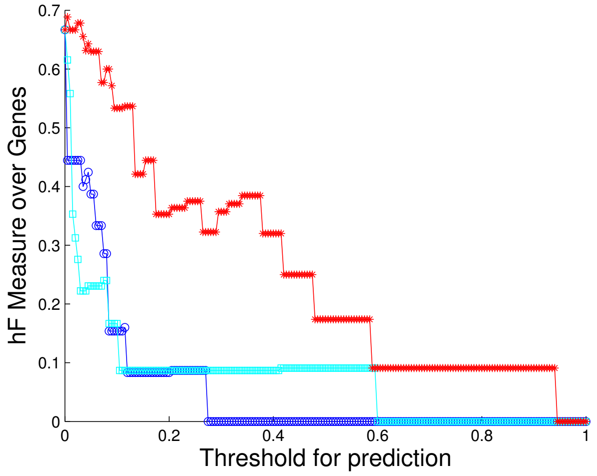

15. **Hierarchy root: GO:0050875, cellular physiological process**
Number of terms in hierarchy = 83
Number of terms predicted = 58
Number of genes predicted = 46

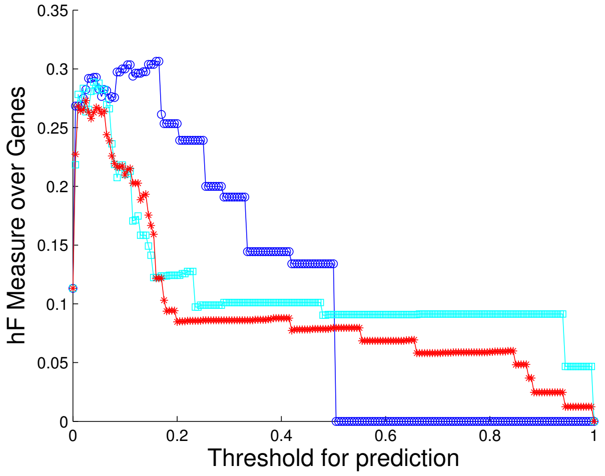

16. **Hierarchy root: GO:0050896, response to stimulus**
    Number of terms in hierarchy = 72
    Number of terms predicted = 39
    Number of genes predicted = 8

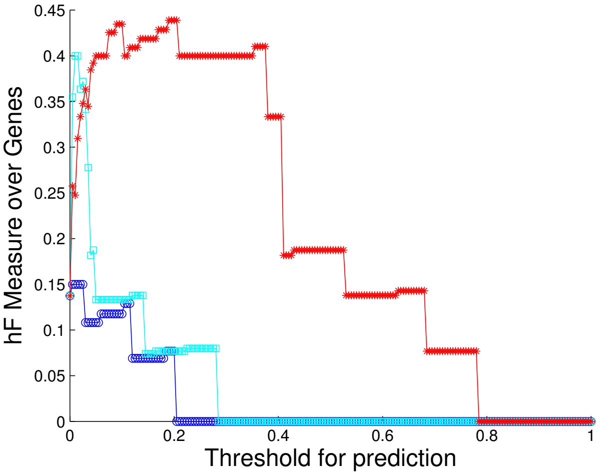

17. **Hierarchy root: GO:0051234, establishment of localization**
    Number of terms in hierarchy = 8
    Number of terms predicted = 5
    Number of genes predicted = 12

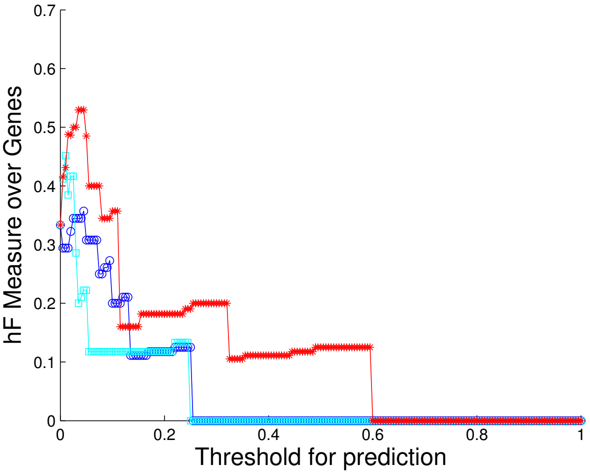

Supplement: Additional file 2 — hF plots for 17 sub-hierarchies in in silico study. This file contains the plots of hF score versus threshold of the three methods for individual sub-hierarchies in the in silico validation study. The root term ID's and names of the root terms, the sizes of sub-hierarchies, numbers of terms and genes predicted within sub-hierarchy are also shown. Colors: HBN (red); BN (light blue); NN (blue). [file 1471-2105-9-350-S2.pdf]
